# Novel tetracyclic structures from the synthesis of thiolactone-isatin hybrids

**DOI:** 10.3762/bjoc.6.78

**Published:** 2010-07-19

**Authors:** Renate Hazel Hans, Hong Su, Kelly Chibale

**Affiliations:** 1 Department of Chemistry and Biochemistry, University of Namibia, Windhoek, Namibia; 2 Department of Chemistry, University of Cape Town, Rondebosch 7701, South Africa; 3 Institute of Infectious Disease and Molecular Medicine, University of Cape Town, Rondebosch 7701, South Africa

**Keywords:** bridged amides, hybrids, isatin, tetracycles, thiolactomycin, thiolactone

## Abstract

A simple and straightforward synthetic approach to potential anti-infective thiolactone-isatin hybrids led to the discovery of novel tetracyclic compounds which bear a macrocylic motif containing an unusual bridged amide bond.

## Introduction

**Figure 1:**
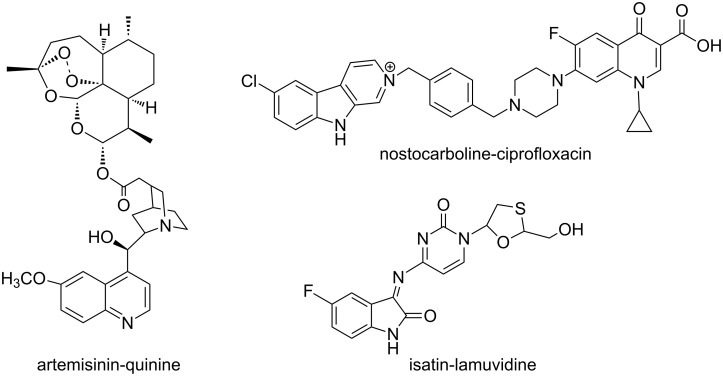
Natural product and natural product-like hybrids.

**Figure 2:**
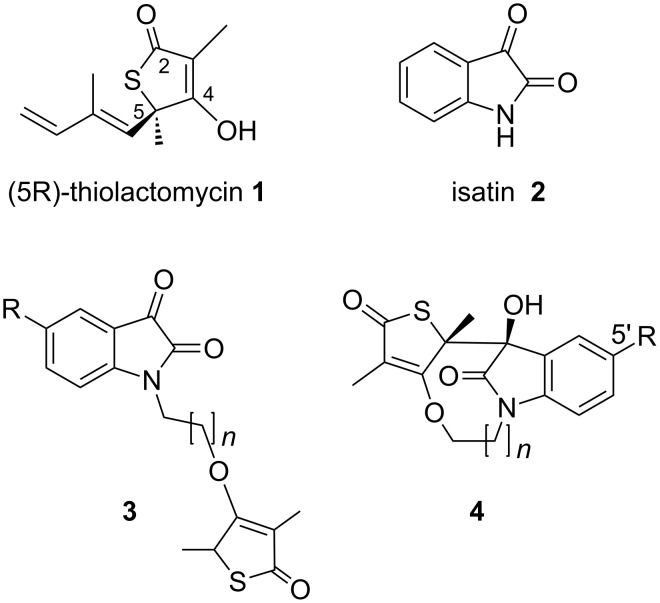
Structures of thiolactomycin (**1**), isatin (**2**), the desired hybrid **3** and tetracycle **4**.

Hybrid structures have been found in nature and their synthesis has become a useful drug discovery strategy for new anti-infective agents [1]. Improved pharmacokinetics, therapeutic and toxicity profiles have been reported for natural and natural product-like hybrid constructs such as the artemisinin-quinine [2], nostocarboline-ciprofloxacin [3] and isatin-lamuvidine [4] (Figure 1). Interest in exploring this approach also derives from the pharmacophore-rich compound library it offers. This precludes the need for large libraries and concomitantly shortens the time needed to identify potential leads [5]. The focus of this study is on the synthesis of hybrids with monomers derived from natural products thiolactomycin (**1**) and isatin (**2**) (Figure 2). The former is a thiolactone antibiotic known for its remarkable selectivity against the condensing enzymes of type II fatty acid synthesis which operate in a number of pathogenic organisms [6–8]. Synthetic methodologies for **1** and a series of racemic and enantiopure thiolactomycin-based analogues have been reported [9–16]. Of particular interest to this project are the structure-activity relationships delineated from *O*-4 modified analogues which reportedly display enhanced antimalarial and promising antitubercular activity [15–16]. The other component of the envisaged hybrid, isatin (**2**) is an intriguing and synthetically versatile scaffold. Moreover, its presence in a number of proprietary molecules and synthetic drugs is noteworthy [17].

**Scheme 1:**
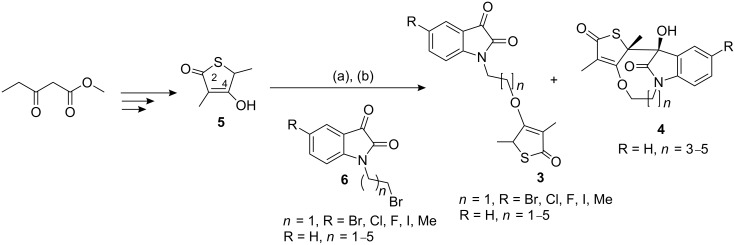
Reagents and conditions: (a) KOH, MeOH, 25 °C, 95–100% (b) **6**, DMF, 60 °C, 9–36%.

Alkylation of **5**, as shown in Scheme 1, is a common route towards diverse thiolactomycin analogues. A literature review revealed that γ-thiolactones are prone to undergo ring-opening polymerization under the basic conditions required to effect alkylation [18] and this has been cited as a likely contributor to the low yields reported in thiolactomycin analogue synthesis. For example, Takabe and co-workers [19] attempted *O*-alkylation of **5** using NaH and CH_3_I at room temperature. Although the reaction proceeded regioselectively, only 9% of the desired C-4 *O*-alkylated product was obtained after a reaction time of 144 h.

We herein report the synthesis of a novel series of thiolactone-isatin hybrids. The envisaged target compounds **3**, as shown in Figure 2, are modeled on **1** but devoid of the hydrophobic side chain represented by the isoprenoid moiety at C-5.

## Results and Discussion

The key intermediate, thiolactone **5** (Scheme 1), was synthesized using a four-step reaction developed by Wang and Salvino [20]. The *N*-alkylated 5-substituted isatin/isatin intermediates **6** were obtained in a one-step reaction in good to excellent yields by the reported procedure [21]. Thus, with **5** and **6** in hand, synthesis of the desired hybrids **3** was subsequently undertaken.

For our synthesis we explored the use of bases of varying strengths, starting with NaH. Although NaH afforded the desired hybrids **3** and traces of the tetracycle **4**, the obtained yields were very poor. Next, the use of the weaker base, K_2_CO_3_, was attempted. Refluxing **5** and **6** in acetone in the presence of 4 equiv of K_2_CO_3_ did not produce **3**. Instead, an unidentified product was isolated from the product mixture, whose ^1^H and ^13^C NMR suggested that it is possibly an aldol product. All efforts were then directed towards a method which utilizes the potassium salt of **5**. The salt was obtained in quantitative yield from the reaction of **5** with an equimolar amount of aqueous potassium hydroxide in MeOH at room temperature. Although no reaction between the potassium salt of **5** and 1.5 equiv of **6** in anhydrous DMF was observed at room temperature, when the temperature was increased to 60 °C, TLC confirmed the consumption of the starting material after stirring at this temperature for 48 h (see Supporting Information File 1 for full experimental data). The difficulties in purification of the crude reaction mixtures may have contributed unfavourably towards the yields obtained for hybrids **3** (Table 1). These yields, although disappointingly low, are in agreement with published reports on thiolactomycin analogues synthesis [15,18,20,22–23]. Of particular interest, however, was the formation of by-products (tetracycles) observed in the reaction of **5** with the *N*-alkylated isatin intermediates **6** bearing the *n* = 3–5 linkers (Table 1). The tetracycle **4** had a slightly lower *R*_f_ than the desired hybrid **3** and was colorless or light yellow compared to the orange-colored hybrid **3**. We initially suspected it to be a regioisomer of **3**, that is, the C-3-alkylated or C-2 *O*-alkylated product consistent with the resonance contributors of the anion of **5**. Lack of regioselectivity in the alkylation reactions of thiolactomycin and its analogues is a common synthetic problem [22–24].

**Table 1:** Synthesis of thiolactone-isatin hybrids **3** and tetracycles **4**.

Compd	R	*n*	(**3**:**4**)^a^	Yield (%)

**3a**	Br	1	100:0	9
**3b**	Cl	1	100:0	13
**3c**	F	1	100:0	14
**3d**	I	1	100:0	10
**3e**	CH_3_	1	100:0	26
**3f**	H	1	100:0	36
**3g**	H	2	100:0	18
**3h**, **4a**	H	3	22:78	45^b^
**3i**, **4b**	H	4	84:16	31^b^
**3j**, **4c**	H	5	71:29	21^b^

^a^Determined from isolated yields; ^b^Combined isolated yields of **3** and **4**.

Interest in the tetracycles **4** was further sparked by the ease and simplicity of their preparation, and the novelty of their architecture as revealed by single X-ray crystal structure analysis. Attractive structural features of **4** include two vicinal quaternary stereocenters and a complex, bicyclic ring system containing a bridged amide bond. Essentially, the amide functionality adopts a planar conformation in normal amides with stability being conferred by the delocalization of the nitrogen lone pair into the C=O bond [25]. The amides in bicyclic bridgehead lactams are distorted from planarity and this severely affects their reactivity and stability [26–31]. Furthermore, the use of bridged amides as scaffolds in medicinal chemistry has been explored [32–37]. Indeed, the enhanced electrophilicity or acylating ability of β-lactams is partially attributed to the distortion from planarity of the amide bond [28,38]. Also present in the structure of the tetracycles **4** is the 3-substituted 3-hydroxyindolin-2-one moiety which features prominently in a number of biologically active natural products [39–41].

**Scheme 2:**
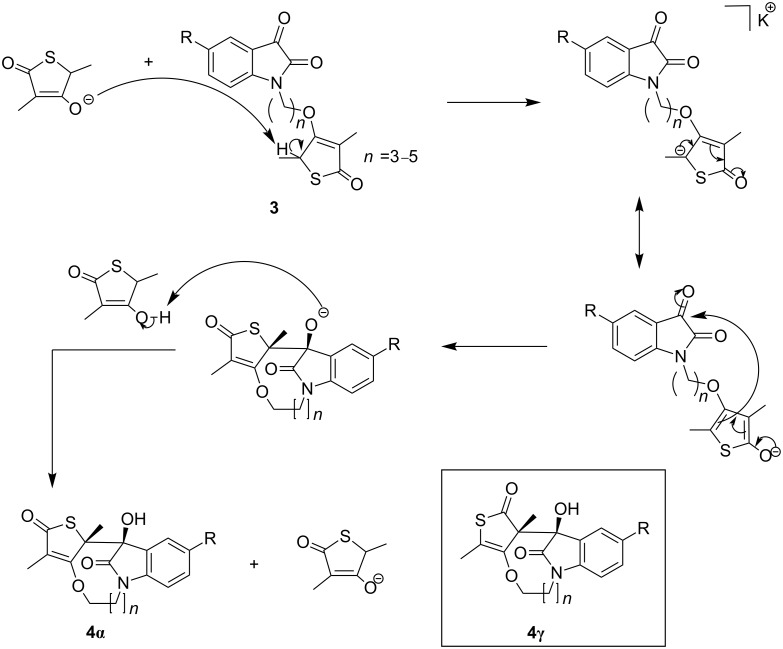
Proposed mechanism for the formation of tetracycles **4**.

The mechanism proposed for the formation of **4** as outlined in Scheme 2, involves an in situ deprotonation at C-5 of the thiolactone ring system by the relatively basic potassium salt of **5**. This is followed by the regioselective, intramolecular addition of the nucleophile to the ketonic carbonyl facilitated by (i) the strong electrophilicity of the α-keto amide group of the isatin scaffold, (ii) the flexible alkane tether (*n* = 3–5) which allows for the proper alignment of the orbitals of the reacting groups and (iii) the rigidity [42] of the isatin and thiolactone ring systems. In the absence of supporting information we speculate that the observed regioselectivity may be due to the thermodynamically more stable product **4α** arising from reaction at the α-position due to a more extended conjugated system relative to the product **4γ** (Scheme 2) that would result from reaction at the γ-position. Scheme 2 also indicates that only one diastereomer of tetracycle **4** is formed as indicated by X-ray crystallographic data. The absence of the other diastereomer may be due to destabilizing steric interactions with the carbonyl group of the amide and the oxygen of the vinyl ether. Similar cyclizations leading to bridged lactams have been reported [43–44].

Synthesis of tetracycles **4** was therefore expanded to obtain a small library for biological testing. Although a model study revealed that prolonged reaction times (120 h) resulted in an increase in tetracycle **4** formation, we were unable to significantly increase the yields of the bridged products despite much longer reaction times (Table 2).

**Table 2:** Synthesis of thiolactone-isatin hybrids **3** and tetracycles **4**.

Compd	R	*n*	(**3**:**4**)^a^	Yield (%)^c^

**4d**	Br	3	11:89	14
**4e**	I	3	10:90	15
**4f**	F	3	19:81	10
**4g**	Cl	3	15:85	14
**4h**	NO_2_	3	30:70	8
**4i**	Cl	4	63:37^b^	5
**4j**	Br	4	67:33^b^	7
**4k**	I	4	60:40^b^	4
**4l**	Cl	5	80:20	6
**4m**	F	5	69:31	7
**4n**	Br	5	73:27	6
**4o**	I	5	70:30	7

^a^Determined from isolated yields; ^b^Ratio determined from crude reaction mixture using ^1^H NMR; ^c^Isolated yields of **4**.

It is noteworthy that **4** did not form with the *n* = 1–2 linked hybrids **3a**–**g** (Table 1). This is most likely due to strain in the transition state. Also notable is the decrease in yield with increasing linker length as evidenced by the differences observed for the *n* = 3–5 linked tetracycles/5′-substituted tetracycles (Table 1 and Table 2). This drawback can most likely be ascribed to entropy factors and stereoelectronic effects [45–48].

**Figure 3:**
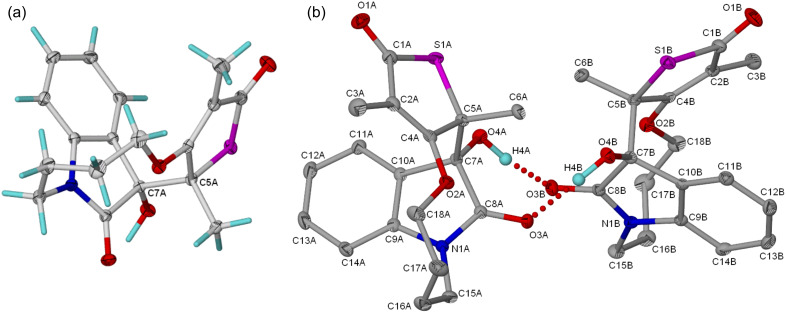
(a) Molecular structure of the tetracycle **4a**. (b) Structure of the dimer of **4a** showing the atomic numbering scheme. Displacement ellipsoids are drawn at 40% probability level for non-hydrogen atoms. All hydrogen atoms are omitted except the hydroxyl hydrogens.

The structures of the tetracycles **4** were unambiguously assigned by single crystal X-ray crystallographic analysis. Single crystals for **4a**–**c** were obtained from MeOH at room temperature and subjected to crystallographic analysis (see Supporting Information File 2 for crystallographic data). Figure 3 shows the molecular structure and atom labeling for **4a**. The crystals are triclinic with space group *P*−1 and contains four molecules per unit cell. The crystal structure furthermore revealed the presence of two crystallographic independent molecules in the unit cell differentiated with the labels A and B in the atom numbering scheme (Figure 3b). Because of similar trends observed in the bond angles and lengths, we have limited our discussion to molecule A. In this structure (Figure 3b) the indole ring is almost planar with C7A and C8A showing deviations of −0.0918(10) and +0.0902(11), respectively from the least square plane. The *syn* relationship between the methyl at C5A and the hydroxyl group at C7A was evident from the torsion angle of −65.81° (14) for O4A-C7A-C5A-C6A. Also noted are the dihedral angles 106.80° (11) and 113.29° (11) for O4A-C7A-C5A and C6A-C5A-C7A, respectively, which shows a distortion from the ideal value (109.47°) for a tetrahedral sp^3^ carbon. Speculatively, this distortion coupled with the elongated C7A-C5A [1.5783(19) Å] bond indicates a repulsive steric interaction between the relatively bulky hydroxyl group at C7A and the adjacent methyl group at C5A.

**Figure 4:**
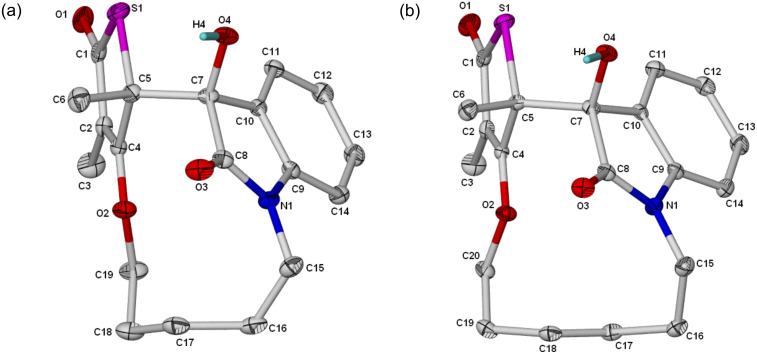
Molecular structure of (a) **4b** and (b) **4c**, with ellipsoidal model of probability level = 35%.

Figure 4 shows the molecular structure atom labeling for **4b**–**c**. Both compounds crystallized in the centrosymmetric monoclinic space group P2_1_/*n* with 4 molecules in the unit cell. Similar to **4a**, the indole moiety is nearly planar with the maximum deviation from the least-squares plane for all the nine atoms in the ring at +0.045(1) and +0.0412(15) for C8, in **4b** and **4c**, respectively.

In recognition of the potential contribution of distorted bridged amides to biological activity as alluded to earlier, the calculation of the Dunitz–Winkler distortion parameters (τ, χ_C_, χ_N_) [25,49–50] for the tetracycles **4a**–**c** was undertaken (Table 3). Compared to planar formamide whose distortion parameters are all 0° [53] compound **4a** with a twist angle τ of 10.4° (Table 3), show a slight distortion from planarity, whereas the amide distortion parameters for **4b**–**c** indicate that the amide bond is close to being planar. It is also known that twisted amides contain pyramidal nitrogen atoms, which makes it a potential site for reaction with protons and electrophiles [25,31]. However, for **4a**–**c** the carbon and nitrogen pyramidality (χ_C_ and χ_N_) and the N-C(=O) and C=O bond lengths (Table 3) all indicate that the bridge amide bond is essentially relaxed.

**Table 3:** Calculated Dunitz-Winkler distortion parameters and amide bond lengths of planar formamide and **4a**–**c**.

Parameter^a^	Formamide (planar)^b^	**4a**^c^	**4b**	**4c**

τ/°	0.0	10.4	5.3	0.6
χ_C_/°	0.0	0.7	3.0	4.5
χ_N_/°	0.0	5.1	1.1	8.9
Bond length/Å C=O	1.193	1.227(17)	1.223(16)	1.223(2)
Bond length/Å N-C(=O)	1.349	1.357(18)	1.355(18)	1.361(3)

^a^[25,49–50]; ^b^[51–53]; ^c^For molecule A.

In summary we have described the straightforward synthesis of novel compounds **3** and **4**. The latter bears quaternary stereogenic centers and a macrocylic motif containing an unusual bridged amide bond. The poor yields obtained are a concern and require further optimization of reaction conditions. A study of the biological activity of the novel structures (**3** and **4**) is currently underway.

## Supporting Information

Supporting information contains full experimental and spectral data for thiolactone **5**, the desired hybrids **3**, tetracycles **4** and the general procedure for the synthesis of the N-alkylated 5-substituted isatin/isatin intermediates **6**. The ^1^H NMR and ^13^C NMR spectra for compounds **3** and **4** are also included.

File 1 Experimental procedures and spectral data for compounds **3**–**6**

File 2 X-ray crystallography data for tetracycles **4a**–**c**
