# Supplementary material for: Novel tetracyclic structures from the synthesis of thiolactone-isatin hybrids
Source: Beilstein J Org Chem. 2010 Jul 19;6:78. doi: 10.3762/bjoc.6.78 (PMC2919273; doi:10.3762/bjoc.6.78)
Supplement: File 1 — Experimental procedures and spectral data for compounds 3–6 [file Beilstein_J_Org_Chem-06-78-s001.pdf]

Supporting Information

for

Novel tetracyclic structures from the synthesis of thiolactone-isatin hybrids

Renate Hazel Hans[1], Hong Su[2] and Kelly Chibale*[2,3]

Address: [1]Department of Chemistry and Biochemistry, University of Namibia, Windhoek, Namibia, [2]Department of Chemistry, University of Cape Town, Rondebosch 7701, South Africa and [3]Institute of Infectious Disease and Molecular Medicine, University of Cape Town, Rondebosch 7701, South Africa

Email: Renate Hans - rhans@unam.na; Hong Su - hong.su@uct.ac.za; Kelly Chibale* - Kelly.Chibale@uct.ac.za

*Corresponding author

Experimental procedures and spectral data for compounds 3–5

General S1

Experimental procedures S2–S16

^{1}H NMR and ^{13}C NMR Spectra S17–S66

References S67

General

All commercially available chemicals used in this project were purchased either from Sigma-Aldrich or Merck in South Africa. With the exception of DMF and MeOH, which were purchased as anhydrous solvents, all solvents used were purified and dried as described in literature [1]. Reactions were monitored by thin layer chromatography (TLC) using Merck F_{254} aluminium-backed silica gel 60 coated plates. Detection of the

spots was either done using the naked eye (especially for the highly coloured isatin derivatives) or by ultraviolet light (254 nm/366 nm). Column chromatography and preparative layer chromatography were carried out on silica gel (Merck Kieselgel 60) and used in the purification of samples.

Proton nuclear magnetic resonance (^{1}H NMR) spectra were recorded in $CDCl_3$, CD_3OD or $(CD_3)_2SO$ on a Varian Gemini (300 MHz) or Varian Unity Spectrometer (400 MHz) with tetramethylsilane (TMS) as internal standard. Carbon-13 nuclear magnetic resonance (^{13}C NMR) spectra were recorded on the same instruments at 75 MHz or 100 MHz. Melting points were determined using a Reichert-Jung Thermovar hot stage microscope and are uncorrected. Infrared (IR) spectra were recorded on a Thermo Nicolette FTIR instrument in the 3800 cm^{-1}–900 cm^{-1} range as chloroform solutions, KBr pellets or on NaCl plates. Microanalyses were performed on a Fisons EA 1108 CHNS-O instrument. High resolution mass spectrometry (HRMS) was performed on a VG70-SEQ (in EI and ESI mode) at the University of Witwatersrand (SA).

X-ray single crystal intensity data were collected on a Nonius Kappa-CCD diffractometer using graphite monochromated MoKα radiation. Temperature was controlled by an Oxford Cryostream cooling system (Oxford Cryostat). The strategy for the data collections was evaluated using the Bruker Nonius "Collect" program. Data were scaled and reduced using DENZO-SMN software [2]. Absorption correction was made empirically by utilizing SADABS program [3]. The structure was solved by direct methods and refined employing full-matrix least-squares with the program SHELXL-97 [4] refining on F^2. Packing diagrams were produced using the program PovRay and graphic interface X-seed [5].

<u>Experimental procedures</u>

4-Hydroxy-3,5-dimethylthiophen-2(5*H*)-one, 5

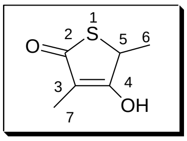

A 10% KOH solution (1.4 g, 25.6 mmol, 2.0 equiv) was added to a stirred solution of thioester (2.8 g, 12.8 mmol, 1.0 equiv) in 16 mL ethanol at 0 °C. The reaction mixture was stirred at room temperature for 4 h. The solvent was then evaporated under reduced pressure, and the residue taken up in water. The resulting mixture was extracted with diethyl ether and the ether layer discarded. The

aqueous layer was acidified to pH 1 with 10% HCl and extracted with EtOAc. The combined organic layer was washed with saturated brine solution and dried over anhydrous Na_2SO_4. Evaporation of the solvent under reduced pressure gave a crude product mixture. Recrystallization from DCM gave the thiolactone as an off-white solid (1.7 g, 60%); R_f (EtOAc:Hex 1:10) 0.36; mp 130–131 °C (lit. mp 128–130 °C)[6]; δ_H (300MHz, CD_3OD) 4.12 (1H, q, *J* 7.1, H-5), 1.66 (3H, s, H-7),1.56 (3H, d, *J* 7.1, H-6); δ_C (75 MHz, CD_3OD) 198.6, 181.2, 110.9, 44.1, 19.6, 7.6

A. General procedure for the synthesis of N-alkylated isatin/5-substituted isatin intermediates 6

Sodium hydride, 60% suspended in mineral oil (1.5 equiv) was added to commercially available isatin/5-substituted isatin (1.0 equiv) in 10 mL of anhydrous DMF at 0 °C. The dibromoalkane (4.0 equiv) was added, the resulting mixture slowly warmed to 25 °C and stirred for 1 h at this temperature. The temperature was then increased to 60 °C and the reaction mixture stirred for 24 h at this temperature. Ice-cold water was added to the orange coloured reaction mixture and the precipitate that formed was filtered, washed with water and recrystallized from MeOH.

B. General procedure for the synthesis of compounds 3a–j and 4a–c

The appropriate *N*-alkylated 5-substituted isatin/isatin **6** (4.11 mmol, 1.5 equiv) and potassium salt of **5** (2.74 mmol, 1.0 equiv) was dissolved in 3 ml anhydrous DMF. The resulting mixture was stirred at 60 °C for 48 h under a N_2 atmosphere. When the reaction was completed, ice-cold water was added to the dark red product mixture. The precipitate so obtained was filtered, washed with water and purified using column chromatography (eluent EtOAc:Hex 3:2). When no precipitate formed upon the addition of ice-cold water, the product mixture was extracted with EtOAc. The combined organic layer was then washed with deionized water to remove DMF and subsequently dried over anhydrous Na_2SO_4. Concentration under reduced pressure afforded the crude product mixture which was subjected to repeated column chromatography to yield the pure product after recrystallization.

5-Bromo-1-[2-(2,4-dimethyl-5-oxo-2,5-dihydro-thiophen-3-yloxy)ethyl]-indole-2,3-dione, (3a)

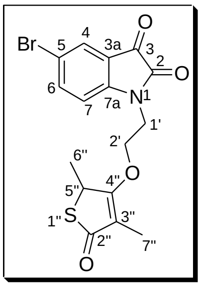

Orange solid (97.7 mg, 9%), m.p. 168–169 °C; R_f (EtOAc:Hex 1:1) 0.28; IRν_{max}(KBr)/cm^{-1} 1748 (C_3=O), 1624 ($C_{2''}$=O), 1603 (C_2=O); δ_H (400 MHz, $CDCl_3$) 7.73 (2H, m, H-4 and H-6), 6.95 (1H, d, *J* 7.5, H-7), 4.57 (1H, m, H-2'a/b), 4.46 (1H, m, H-2'a/b), 4.08 (3H, m, H-5" and H-1'), 1.76 (3H, d, *J* 1.6, H-7"), 1.49 (3H, d, *J* 6.8, H-6"); δ_C (100MHz, $CDCl_3$) 194.5, 182.0, 176.4, 157.7, 149.5, 140.5, 128.5, 118.8, 117.1, 115.3, 112.0, 68.2, 41.7, 40.5, 19.7, 9.1; HRMS(EI) found *m/z* 394.98277 for $C_{16}H_{14}O_4NSBr$, requires

394.98269; Anal. Calc. for $C_{16}H_{14}O_4NSBr$: C, 48.50%; H, 3.56%; N, 3.53%; S, 8.09%. Found: C, 48.40%; H, 3.45%; N, 3.70%; S, 7.70%.

5-Chloro-1-[2-(2,4-dimethyl-5-oxo-2,5-dihydro-thiophen-3-yloxy)-ethyl]-1*H*-indole-2,3-dione, (3b)

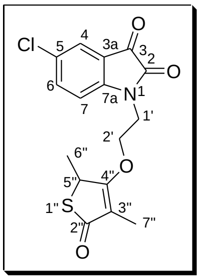

Orange solid (125 mg, 13%); mp 270–272 °C; R_f (EtOAc:Hex 1:1) 0.23; IRν_{max}(KBr)/cm^{-1} 1743 (C_3=O), 1633 ($C_{2''}$=O), 1610 (C_2=O); δ_H (400MHz, $CDCl_3$) 7.58 (2H, m, H-6 and H-4), 7.00 (1H, d, *J* 8.0, H-7), 4.56 (1H, m, H-2'a/b), 4.46 (1H, m, H-2'a/b), 4.08 (3H, m, H-5" and H-1'), 1.76 (3H, d, *J* 1.2, H-7"), 1.25 (3H, d, *J* 7.2, H-6"); δ_C (100MHz, $CDCl_3$) 195.1, 181.5, 176.4, 157.8, 149.1, 137.7, 130.1, 125.6, 118.4, 115.3, 111.7, 68.2, 41.7, 40.5, 19.7, 9.1; HRMS(EI) found *m/z* 350.99468, $C_{16}H_{14}O_4NSCl$, requires 351.03321; Anal. Calc. for $C_{16}H_{14}O_4NSCl$: C, 54.62%; H, 4.01%; N, 3.98%; S, 9.11%. Found: C, 54.41%; H, 3.88; N, 3.80%; S, 8.89%.

1-[2-(2,4-Dimethyl-5-oxo-2,5-dihydro-thiophen-3-yloxy)-ethyl]-5-fluoro-1*H*-indole-2,3-dione, (3c)

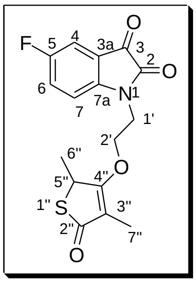

Orange solid (129 mg, 14%), mp 132–133 °C; R_f (EtOAc:Hex 1:1) 0.19; IRν_{max}(KBr)/cm^{-1} 1740 (C_3=O), 1641 ($C_{2''}$=O), 1620 (C_2=O); δ_H (400MHz, $CDCl_3$) 7.34 (2H, m, H-4 and H-6), 7.02 (1H, dd, *J* 4.0 and 9.6, H-7), 4.57 (1H, m, H-2'a/b), 4.46 (1H, m, H-2'a/b), 4.09 (3H, m, H-5" and H-1'), 1.76 (3H, d, *J* 1.2, H-7"), 1.49 (3H, d, *J* 6.8, H-6"); δ_C (100MHz, $CDCl_3$) 195.1, 181.9, 176.5, 160.7, 158.2, 146.8, 124.7, 118.3, 115.3, 112.7, 111.6, 68.2, 41.7, 40.5, 19.7, 9.0; HRMS(EI) found *m/z* 335.06248 for $C_{16}H_{14}O_4NSF$, requires 335.06276; Anal. Calc. for $C_{16}H_{14}O_4NF$: C, 57.30%; H, 4.21%; N, 4.18%; S, 9.56%. Found: C, 57.13%; H, 4.06%; N, 3.72%; S, 9.22%.

1-[2-(2,4-Dimethyl-5-oxo-2,5-dihydro-thiophen-3-yloxy)-ethyl]-5-iodo-1*H*-indole-2,3-dione, (3d)

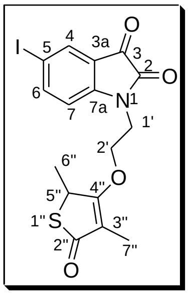

Orange solid (121 mg, 10%); mp 196 °C; R_f (EtOAc:Hex 1:1) 0.26; IRν_{max}(KBr)/cm^{-1} 1740 (C_3=O), 1623 ($C_{2''}$=O), 1600 (C_2=O); δ_H (400MHz, $CDCl_3$) 7.92 (2H, m, H-4 and H-6), 6.84 (1H, d, *J* 8.8, H-7), 4.56 (1H, m, H-2'a/b), 4.45 (1H, m, H-2'a/b), 4.08 (3H, m, H-5" and H-1'), 1.77 (3H, d, *J* 1.2, H-7"), 1.49 (3H, d, *J* 7.2, H-6"); δ_C (100MHz, $CDCl_3$) 195.1, 182.5, 178.4, 169.2, 157.4, 150.1, 146.3, 134.2, 119.1, 112.4, 86.4, 68.2, 41.7, 40.4, 19.7, 9.1; HRMS(EI) found *m/z* 442.97039, $C_{16}H_{14}O_4NSI$, requires 442.96883; Anal. Calc. for $C_{16}H_{14}O_4NSI$: C, 43.35%; H, 3.18%; N, 3.16%; S, 7.23%. Found: C, 42.95%; H, 3.29%; N, 2.82%; S, 6.70%.

1-[2-(2,4-Dimethyl-5-oxo-2,5-dihydro-thiophen-3-yloxy)-ethyl]-5-methyl-1*H*-indole-2,3-dione, (3e)

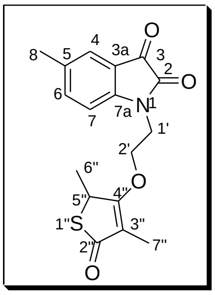

Orange solid (236 mg, 26%), mp 130 °C; R_f (EtOAc:Hex 1:1) 0.23; IRν_{max}(KBr)/cm^{-1} 1740 (C_3=O), 1643 ($C_{2''}$=O), 1619 (C_2=O); δ_H (400MHz, $CDCl_3$) 7.41 (2H, m, H-6 and H-4), 6.91 (1H, d, *J* 8.0, H-7), 4.56 (1H, m, H-2'a/b), 4.46 (1H, m, H-2'a/b), 4.07 (3H, m, H-5" and H-1'), 2.35 (3H, s, H-8), 1.76 (3H, d, *J* 1.6, H-7"), 1.48 (3H, d, *J* 6.8, H-6"); δ_C (100MHz, $CDCl_3$) 195.3, 182.8, 176.6, 158.6, 148.6, 138.7, 134.1, 126.0, 117.6, 115.0, 110.1, 68.2, 41.7, 40.3, 20.6, 19.6, 9.0; HRMS(EI) found *m/z* 331.08822 for $C_{17}H_{17}O_4NS$ requires 331.08783; Anal. Calc. for $C_{17}H_{17}O_4NS$: C, 61.61%; H, 5.17%; N, 4.23%; S, 9.68%. Found: C, 61.51%; H, 5.20%; N, 4.04%; S, 9.35%.

1-[2-(2,4-Dimethyl-5-oxo-2,5-dihydro-thiophen-3-yloxy)-ethyl]-1*H*-indole-2,3-dione, (3f)

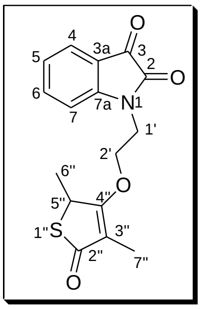

Orange solid (258 mg, 36%); mp 54 °C; R_f (EtOAc:Hex 1:1) 0.18; IRν_{max}(KBr)/cm^{-1} 1740 (C_3=O), 1644 ($C_{2''}$=O), 1611 (C_2=O); δ_H (400MHz, $CDCl_3$) 7.62 (2H, m, H-4 and H-6), 7.16 (1H, td, *J* 0.9 and 7.8, H-5), 7.03 (1H, d, *J* 7.8, H-7), 4.58 (1H, m, H-2'a/b), 4.47 (1H, m, H-2'a/b), 4.09 (3H, m, H-5" and H-1'), 1.75 (3H, d, *J* 1.5, H-7"), 1.48 (3H, d, *J* 6.6, H-6"); δ_C (100MHz, $CDCl_3$) 195.2, 182.5, 176.6, 158.4, 150.8, 138.3, 125.7, 124.2, 117.6, 115.1, 110.3, 68.2, 41.8, 40.3, 19.6, 9.0; HRMS(EI) found *m/z* 317.0722 for $C_{16}H_{15}O_4NS$ requires 317.07218; Anal Calc. for $C_{16}H_{15}O_4NS$: C, 60.55%; H, 4.76%; N, 4.41%; S, 10.10%. Found: C, 60.38%; H, 4.75%; N, 4.01%; S, 9.73%.

1-[3-(2,4-Dimethyl-5-oxo-2,5-dihydro-thiophen-3-yloxy)-propyl]-1*H*-indole-2,3-dione, (3g)

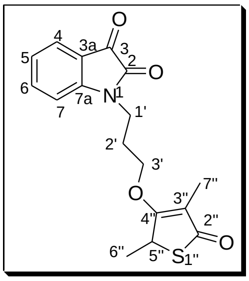

Orange solid (161 mg, 18%); mp 150–151 °C; R_f (EtOAc:Hex 1:1) 0.24; IRν_{max}(KBr)/cm^{-1} 1736 (C_3=O), 1663 ($C_{2''}$=O), 1612 (C_2=O); δ_H (400MHz, $CDCl_3$) 7.62 (2H, m, H-6 and H-4), 7.15 (1H, td, *J* 0.9 and 7.6, H-5), 6.92 (1H, d, *J* 7.6, H-7), 4.39 (1H, m, H-3'a/b), 4.27 (1H, m, H-3'a/b), 4.15 (1H, qd, *J* 1.2 and 7.2, H-5"), 3.93 (2H, t, *J* 6.8, H-1'), 2.18 (2H, m, H-2'), 1.82 (3H, d, *J* 1.2, H-7"), 1.57 (3H, d, *J* 7.2, H-6"); δ_C (100MHz, $CDCl_3$) 195.5, 183.0, 177.3, 158.4, 150.6, 138.5, 125.7, 124.0, 117.7, 114.8, 109.7, 68.3, 41.9, 37.0, 28.0, 19.8, 9.0; HRMS(EI) found *m/z* 331.08695 for $C_{17}H_{17}O_4NS$ requires 331.08783; Anal Calc. for $C_{17}H_{17}O_4NS$: C, 61.67%; H, 5.17%; N, 4.23%; S, 9.68%. Found: C, 61.63%; H, 5.05%; N, 3.99%; S, 9.19%.

1-[4-(2,4-Dimethyl-5-oxo-2,5-dihydro-thiophen-3-yloxy)-butyl]-1*H*-indole-2,3-dione, (3h)

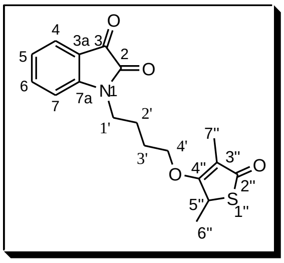

Orange solid (91.6 mg, 10%); mp 108 °C; R_f (EtOAc:Hex 1:1) 0.18; IRν_{max}(KBr)/cm^{-1} 1733 (C_3=O), 1639 ($C_{2''}$=O), 1606 (C_2=O); δ_H (300MHz, $CDCl_3$) 7.60 (2H, m, H-6 and H-4), 7.13

(1H, td, *J* 0.9 and 7.8, H-5), 6.90 (1H, d, *J* 7.8, H-7), 4.35 (1H, m, H-4’a/b), 4.22 (1H, m, H-4’a/b), 4.14 (1H, qd, *J* 0.9 and 6.9, H-5”), 3.81 (2H, t, *J* 6.9, H-1’), 1.86 (4H, m, H-2’ and H-3’), 1.80 (3H, d, *J* 0.9, H-7”), 1.55 (3H, d, *J* 6.9, H-6”); δ_C (75MHz, $CDCl_3$) 195.7, 183.2, 177.7, 158.3, 150.6, 138.3, 125.6, 123.9, 117.6, 114.5, 109.9, 70.2, 41.9, 39.5, 27.1, 23.6, 19.8, 9.0; HRMS(EI) found *m/z* 345.1029 for $C_{18}H_{19}O_4NS$ requires 345.10348; Anal Calc. for $C_{18}H_{19}O_4NS$: C, 62.59%; H, 5.54%; N, 4.06%; S, 9.28%. Found: C, 62.20%; H, 5.52%; N, 4.27%; S, 8.96%.

1-[5-(2,4-Dimethyl-5-oxo-2,5-dihydro-thiophen-3-yloxy)-pentyl]-1*H*-indole-2,3-dione, (3i)

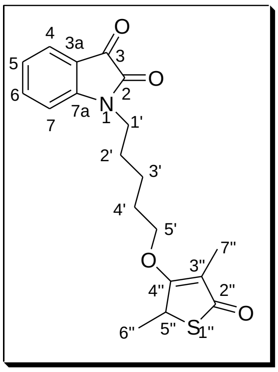

Orange solid (217 mg, 22%); mp 77–79 °C; R_f (EtOAc:Hex 1:1) 0.36; IRν_{max}(KBr)/cm^{-1} 1732 (C_3=O), 1670 ($C_{2''}$=O), 1620 (C_2=O); δ_H (400MHz, $CDCl_3$) 7.59 (2H, m, H-6 and H-4), 7.12 (1H, td, *J* 0.9 and 7.6, H-5), 6.89 (1H, d, *J* 8, H-7), 4.30 (1H, m, H-5’a/b), 4.13 (2H, m, H-5’a/b and H-5”), 3.77 (2H, t, *J* 7.2, H-1’), 1.80 (7H, m, H-7”, H-2’ and H-4’), 1.54, (5H, m, H-6” and H-3’); δ_C (100MHz, $CDCl_3$) 195.4, 183.3, 177.9, 158.2, 150.8, 138.3, 125.6, 123.8, 117.6, 114.3, 110.0, 70.8, 41.9, 39.8, 29.4, 26.9, 23.1, 19.8, 9.0; HRMS(EI) found *m/z* 359.11842 for $C_{19}H_{21}O_4NS$ requires 359.11913; Anal Calc. for $C_{19}H_{21}O_4NS$: C, 63.49%; H, 5.89%; N, 3.90%; S, 8.92%. Found: C, 63.43%; H, 5.94%; N, 3.40%; S, 7.86%.

1-[6-(2,4-Dimethyl-5-oxo-2,5-dihydro-thiophen-3-yloxy)-hexyl]-1H-indole-2,3-dione, (3j)

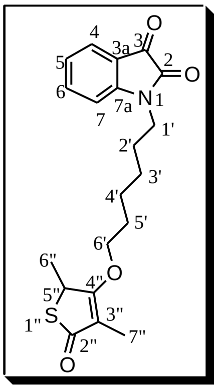

Orange oil (154 mg, 15%); R_f (EtOAc:Hex 1:1) 0.31; IRν_{max}($CHCl_3$)/cm^{-1} 1739 (C_3=O), 1675 ($C_{2''}$=O), 1611 (C_2=O); δ_H (300MHz, $CDCl_3$) 7.57 (2H, m, H-4 and H-6), 7.09 (1H, td, *J* 0.9 and 7.4, H-5), 6.88 (1H, d, *J* 7.8, H-7), 4.27 (1H, m, H-6’a/b), 4.13 (2H, m, H-6’a/b and H-5”), 3.72 (2H, t, *J* 7.5, H-1’), 1.79 (3H, d, *J* 0.9, H-7”), 1.76-1.68 (4H, m, H-2’ and H-5’), 1.54 (3H, d, *J* 6.6, H-6”), 1.41-1.50 (4H, m, H-3’ and H-4’); δ_C (75MHz, $CDCl_3$) 195.7, 183.4, 178.0, 158.1, 150.8, 138.2, 125.4, 123.6, 117.5, 114.0, 110.0, 71.0, 41.9, 39.9, 29.7, 27.1, 26.4, 25.3, 19.8, 9.0;

HRMS(EI) found *m/z* 373.1346 for $C_{20}H_{23}O_4NS$ requires 373.13478; Anal. Calc. for $C_{20}H_{23}O_4NS$: C, 64.32%; H, 6.21%; N, 3.75%; S, 8.59%. Found: C, 63.64%; H, 6.46%; N, 3.65%; S, 7.24%.

The conditions employed for the preparation of compound **4a–o** are those described in general procedure B. However, the reaction time for compounds **4d–p** was increased from 48 h to 120 h.

Compound 4a (R = H, n = 3)

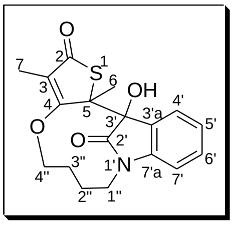

Yellow crystalline solid (333 mg, 35%); mp 184–185 °C; R_f (EtOAc:Hex 1:1) 0.29; IRν_{max}(KBr)/cm^{-1} 3311 (OH), 1700 (C_2=O), 1620 ($C_{2'}$=O), 1468 (Ar C=C); δ_H (400MHz, $CDCl_3$) 7.80 (1H, dd, *J* 1.2 and 7.6, H-4’), 7.28 (1H, td, *J* 1.2 and 8.0, H-6’), 6.96 (1H, td, *J* 0.8 and 7.6, H-5’), 6.78 (1H, d, *J* 8.0, H-7’), 4.59 (1H, m, H-4”a/b), 4.29 (1H, m, H-1”a/b), 3.55 (1H, m, H-4”a/b), 3.38 (1H, dt, *J* 3.6 and 14.4, H-1”a/b), 3.35 (1H, s, OH), 2.08-1.86 (4H, m, H-2” and H-3”), 1.99 (3H, s, H-7), 1.60 (3H, s, H-6); δ_C (100MHz, $CDCl_3$) 195.3, 176.3, 175.4, 141.7, 130.3, 127.5, 125.8, 122.5, 110.9, 108.4, 78.8, 74.4, 66.1, 39.4, 28.3, 26.7, 19.5, 9.3; HRMS(EI) found *m/z* 345.1030 for $C_{18}H_{19}O_4NS$ requires 345.10348; Anal Calc. for $C_{18}H_{19}O_4NS$: C, 62.59%; H, 5.54%; N, 4.06%; S, 9.28%. Found: C, 62.48%; H, 5.09%; N, 3.70%; S, 9.01%.

Compound 4b (R = H, n = 4)

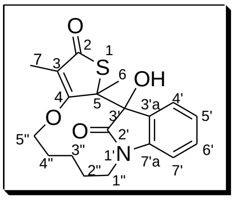

Light yellow crystalline solid (85.3 mg, 9%); mp 214–215 °C; R_f (EtOAc:Hex 1:1) 0.38; IRν_{max}(KBr)/cm^{-1} 3380 (OH), 1695 (C_2=O), 1622 ($C_{2'}$=O), 1471 (Ar C=C); δ_H (400MHz, $CDCl_3$) 7.80 (1H, dd, *J* 1.2 and 7.6, H-4’), 7.28 (1H, td, *J* 1.2 and 8.0, H-6’), 6.96 (1H, td, *J* 0.8 and 7.6, H-5’), 6.78 (1H, d, *J* 8.0, H-7’), 4.59 (1H, m, H-5”a/b), 4.29 (1H, m, H-1”a/b), 3.45 (1H, m, H-5”a/b), 3.29 (1H, br. s, OH), 3.26 (1H, dt, *J* 3.2 and 14.0, H-1”a/b), 1.96 (3H, s, H-7), 1.91–1.72 (4H, m, H-2” and H-4”), 1.60 (3H, s, H-6), 1.44 (2H, m, H-3”); δ_C (100MHz, $CDCl_3$) 195.7, 176.3, 175.2, 142.3, 130.5, 127.8, 125.4, 122.9, 110.8, 107.9, 78.3, 67.7, 66.0, 40.8, 28.5, 23.2, 19.7, 19.5, 9.7; HRMS(EI) found m/z 359.1186 for $C_{19}H_{21}O_4NS$

requires 359.11913; Anal Calc. for $C_{19}H_{21}O_4NS$: C, 63.49%; H, 5.89%; N, 3.90%; S, 8.92%. Found: C, 63.02%; H, 6.08%; N, 2.98%; S, 9.05%.

Compound 4c (R = H, *n* = 5)

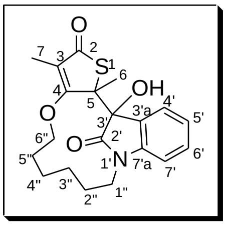

Yellow crystalline solid (61.4 mg, 6%); mp 198 °C; R_f (EtOAc:Hex 1:1) 0.40; IRν_{max}(KBr)/cm^{-1} 3412 (OH), 1697 (C_2=O), 1630 ($C_{2'}$=O), 1471 (Ar C=C); δ_H (300MHz, $CDCl_3$) 7.86 (1H, dd, *J* 1.5 and 7.8, H-4'), 7.28 (1H, td, *J* 1.2 and 7.8, H-6'), 6.94 (1H, td, *J* 0.9 and 7.8, H-5'), 6.83 (1H, d, *J* 7.8, H-7'), 4.26-4.10 (3H, m, H-1"a/b and H-6"), 3.28 (1H, m, H-1"a/b), 3.18 (1H, br. s, OH), 2.04 (3H, s, H-7), 1.98-1.81 (3H, m, H-2"a/b and H-5"), 1.67 (3H, s, H-6), 1.62-1.30 (5H, m, H-2"a/b, H-3" and H-4"); δ_C (75MHz, $CDCl_3$) 195.6, 176.2, 175.2, 144.0, 130.5, 127.2, 125.1, 122.6, 111.7, 108.4, 77.4, 72.5, 65.4, 39.2, 26.0, 24.1, 23.8, 22.6, 21.2, 9.7; HRMS(EI) found *m/z* 373.1341 for $C_{20}H_{23}O_4NS$ requires 373.13478; Anal Calc. for $C_{20}H_{23}O_4NS$: C, 64.32%; H, 6.21%; N, 3.75%; S, 8.59%. Found: C, 64.31%; H, 6.13%; N, 3.70%; S, 8.66%.

Compound 4d (R = Br, *n* = 3)

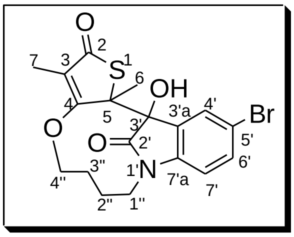

Off-white amorphous powder (158 mg, 14%); mp 156–157 °C; R_f (EtOAc:Hex, 1:1) 0.27; IR ν_{max}(KBr)/cm^{-1} 3416 (OH), 1705 (ester C=O), 1618 (amidic C=O), 1482 (Ar C=C); δ_H (400MHz, $CDCl_3$) 7.93 (1H, d, *J* 1.8, H-4'), 7.43 (1H, dd, *J* 2.1 and 8.4, H-6'), 6.67 (1H, d, *J* 8.4, H-7'), 4.63 (1H, m, H-4"a/b), 4.32 (1H, m, H-1"a/b), 3.59 (1H, m, H-4"a/b), 3.40 (1H, dt, *J* 3.6 and 14.4, H-1"a/b), 3.18 (1H, br. s, OH), 2.08–1.87 (4H, m, H-2" and H-3"), 2.00 (3H, s, H-7), 1.68 (3H, s, H-6); δ_C (100MHz, $CDCl_3$) 194.4, 185.1, 175.0, 156.0, 133.2, 129.4, 129.2, 115.2, 111.1, 109.7, 78.7, 74.5, 65.9, 39.5, 28.3, 26.7, 19.8, 9.5; HRMS(ESI) found *m/z* 424.02123 $[M+H]^+$ for $C_{18}H_{18}O_4NSBr$ requires 423.01399; Anal Calc. for $C_{18}H_{18}O_4NSBr$: C, 50.95%; H, 4.28%; N, 3.30%; S, 7.56%. Found: C, 50.36%; H, 4.45%; N, 2.96%; S, 7.77%.

Compound 4e (R = I, *n* = 3)

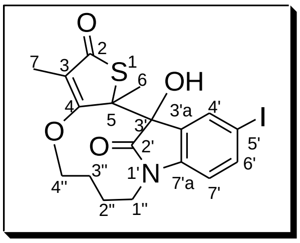

Light yellow solid (188 mg, 15%); mp 205–206 °C; R_f (EtOAc:Hex 1:1) 0.29; IRν_{max}(KBr)/cm^{-1} 3439 (OH), 1715 (C_2=O), 1631 ($C_{2'}$=O), 1480 (Ar C=C); δ_H (400MHz, $CDCl_3$) 8.09 (1H, d, *J* 2.0, H-4'), 7.62 (1H, dd, *J* 2.0 and 8.4, H-6'), 6.58 (1H, d, *J* 8.4, H-7'), 4.62 (1H, m, H-4"a/b), 4.27 (1H, m, H-1"a/b), 3.55 (1H, m, H-4"a/b), 3.48 (1H, br. s, OH), 3.36 (1H, dt, *J* 3.2 and 14.8, H-1"a/b), 2.07–1.84 (4H, m, H-2" and H-3"), 1.97 (3H, s, H-7), 1.66 (3H, s, H-6); δ_C (100MHz, $CDCl_3$) 194.4, 175.8, 175.1, 141.4, 139.1, 134.6, 129.8, 111.1, 110.3, 84.8, 78.7, 74.5, 65.9, 39.5, 28.3, 26.7, 19.8, 9.5; HRMS(ESI) found *m/z* 472.00759 $[M+H]^+$ for $C_{18}H_{18}O_4NSI$ requires 471.00013; Anal Calc. for $C_{18}H_{18}O_4NSI$: C, 45.87%; H, 3.85%; N, 2.97%; S, 6.80%. Found: C, 45.62%; H, 3.34%; N, 2.06%; S, 6.66%.

Compound 4f (R = F, *n* = 3)

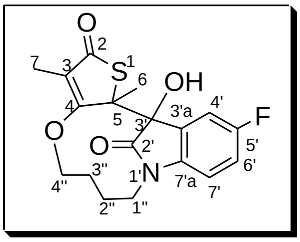

Light yellow solid (95.2 mg, 10%); mp 157 °C; R_f (EtOAc:Hex 1:1) 0.26; IRν_{max}(KBr)/cm^{-1} 3306 (OH), 1701 (C_2=O), 1625 ($C_{2'}$=O), 1489 (Ar C=C); δ_H (400MHz, $CDCl_3$) 7.59 (1H, dd, *J* 2.8 and 8.0, H-4'), 7.01 (1H, ddd, *J* 2.4, 8.6 and 8.8, H-6'), 6.73 (1H, dd, *J* 4.0 and 8.4, H-7'), 4.63 (1H, m, H-4"a/b), 4.31 (1H, m, H-1"a/b), 3.57 (1H, m, H-4"a/b), 3.45 (1H, br. s, OH), 3.39 (1H, dt, *J* 3.2 and 14.4, H-1"a/b), 2.09–1.83 (4H, m, H-2" and H-3"), 1.99 (3H, s, H-7), 1.66 (3H, s, H-6); δ_C (100MHz, $CDCl_3$) 194.4, 177.2, 175.1, 159.8, 147.1, 137.6, 116.7, 114.6, 111.0, 108.9, 78.8, 74.5, 66.0, 39.6, 28.4, 26.7, 19.7, 9.4; HRMS(ESI) found *m/z* 364.10153 $[M+H]^+$ for $C_{18}H_{18}O_4NSF$ requires 363.09406; Anal Calc. for $C_{18}H_{18}O_4NSF$: C, 59.49%; H, 4.99%; N, 3.85%; S, 8.82%. Found: C, 59.37%; H, 4.88%; N, 3.21%; S, 8.72%.

Compound 4g (R = Cl, *n* =3)

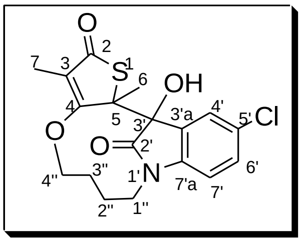

Light yellow solid (151 mg, 14%); mp 208–210 °C; R_f (EtOAc:Hex 1:1) 0.24; IRν_{max}(KBr)/cm^{-1} 3415 (OH), 1707 (C_2=O), 1618 ($C_{2'}$=O), 1484 (Ar C=C); δ_H (400MHz, DMSO-d_6) 7.54 (1H, d, *J* 2.4, H-4'), 7.37 (1H, dd, *J* 2.4 and 8.8, H-6'), 7.04 (1H, d, *J* 8.4, H-7'), 6.86 (1H, br. s, OH), 4.65 (1H, br. dd, *J* 4.0 and 10.0, H-4"a/b), 4.07 (1H, m, H-1"a/b), 3.53 (1H, m, H-4"a/b), 3.46 (1H, m, H-1"a/b), 1.98-1.59 (4H, m, H-2" and H-3"), 1.89 (3H, s, H-7), 1.56 (3H, s, H-6); δ_C (100MHz, DMSO-d_6) 193.8, 175.8, 175.1, 140.6, 130.3, 129.6, 125.1, 124.8, 110.2, 109.8, 77.7, 74.7, 65.6, 48.5, 27.8, 25.9, 19.8, 8.8; HRMS(ESI) found *m/z* 380.07214 $[M+H]^+$ for $C_{18}H_{18}O_4NSCl$ requires 379.06451; Anal Calc. for $C_{18}H_{18}O_4NSCl$: C, 56.91%; H, 4.78%; N, 3.69%; S, 8.44%. Found: C, 55.91%; H, 4.68%; N, 3.49%; S, 8.37%.

Compound 4h (R = NO_2, *n* = 3)

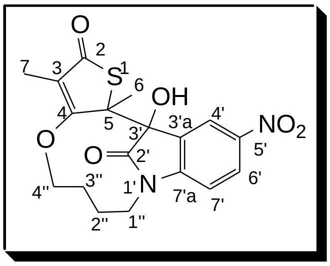

Light yellow crystalline solid (83.5 mg, 8%); mp 215–217 °C; R_f (EtOAc:Hex 1:1) 0.14; IRν_{max}(KBr)/cm^{-1} 3293 (OH), 1739 (C_2=O), 1605 ($C_{2'}$=O), 1523 (Ar C=C), 1335/1523 (Asym./sym. NO_2 str.); δ_H (400MHz, DMSO-d_6) 7.54 (1H, d, *J* 2.4, H-4'), 7.37 (1H, dd, *J* 2.4 and 8.8, H-6'), 7.04 (1H, d, *J* 8.4, H-7'), 6.86 (1H, br. s, OH), 4.65 (1H, m, H-4"a/b), 4.07 (1H, m, H-1"a/b), 3.53 (1H, m, H-4"a/b), 3.46 (1H, m, H-1"a/b), 2.01-1.61 (4H, m, H-2" and H-3"), 1.89 (3H, s, H-7), 1.56 (3H, s, H-6); δ_C (100MHz, DMSO-d_6) 193.4, 175.9, 175.3, 148.0, 141.3, 129.1, 127.0, 119.6, 109.9, 109.1, 77.2, 74.8, 65.5, 39.3, 27.8, 26.0, 19.8, 8.8; HRMS(ESI) found *m/z* 391.09609 $[M+H]^+$ for $C_{18}H_{18}O_6N_2S$ requires 390.08856; Anal Calc. for $C_{18}H_{18}O_6N_2S$: C, 55.38%; H, 4.86%; N, 7.18%; S, 8.21%. Found: C, 55.74%; H, 4.86%; N, 6.29%; S, 8.45%.

Compound 4i (R = Cl, *n* = 4)

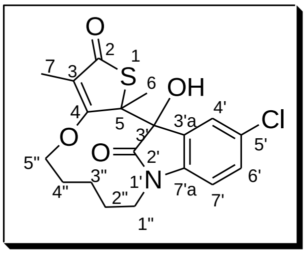

Light yellow solid (57.8 mg, 5%); mp 200–201 °C; R_f (EtOAc:Hex 2:3) 0.32; IRν_{max}(KBr)/cm^{-1} 3287 (OH), 1701 (C_2=O), 1607 ($C_{2'}$=O), 1482 (Ar C=C); δ_H (300MHz, $CDCl_3$) 7.85 (1H, d, *J* 2.1, H-4'), 7.29 (1H, dd, *J* 2.4 and 8.4, H-6'), 6.74 (1H, d, *J* 8.1, H-7'), 4.51 (1H, m, H-5"a/b), 4.17 (1H, m, H-1"a/b), 3.50 (1H, m, H-5"a/b) 3.33 (1H, br. s, OH), 3.26 (1H, dt, *J* 3.0 and 14.1, H-1"a/b), 1.98 (3H, s, H-7), 1.96–1.71 (4H, m, H-2" and H-4"), 1.68 (3H, s, H-6), 1.45 (2H, m, H-3"); δ_C (75MHz, $CDCl_3$) 194.9, 175.7, 174.8, 140.9, 130.4, 129.4, 128.4, 126.0, 112.1, 108.7, 78.2, 67.8, 65.8, 41.0, 28.4, 23.1, 19.8, 19.5, 9.8; HRMS(ESI) found *m/z* 394.08725 $[M+H]^+$ for $C_{19}H_{20}NO_4SCl$ requires 393.08016; Anal Calc. for $C_{19}H_{20}NO_4SCl$: C, 57.94%; H, 5.12%; N, 3.56%; S, 8.14%. Found: C, 57.45%; H, 5.10%; N, 3.39%; S, 8.04%.

Compound 4j (R = Br, *n* = 4)

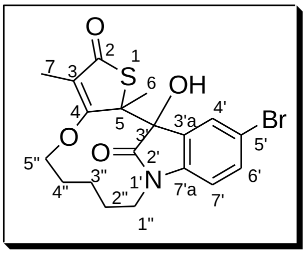

Light yellow solid (88.6 mg, 7%); mp 203–205 °C; R_f (EtOAc:Hex 2:3) 0.34; IRν_{max}(KBr)/cm^{-1} 3321 (OH), 1706 (C_2=O), 1604 ($C_{2'}$=O), 1476 (Ar C=C); δ_H (300MHz, $CDCl_3$) 7.98 (1H, d, *J* 2.4, H-4'), 7.44 (1H, dd, *J* 1.8 and 8.1, H-6'), 6.69 (1H, d, *J* 8.1, H-7'), 4.50 (1H, m, H-5"a/b), 4.15 (1H, m, H-1"a/b), 3.46-3.54 (2H, m, H-5"a/b and OH), 3.25 (1H, dt, *J* 3.0 and 14.0, H-1"a/b), 1.97 (3H, s, H-7), 1.95–1.71 (4H, m, H-2" and H-4"), 1.68 (3H, s, H-6), 1.46 (2H, m, H-3"); δ_C (75MHz, $CDCl_3$) 194.8, 175.9, 174.8, 141.4, 133.3, 129.7, 128.6, 115.5, 112.1, 109.2, 78.2, 67.8, 65.8, 40.9, 28.4, 23.1, 19.8, 19.5, 9.8; HRMS(ESI) found *m/z* 438.03618 $[M+H]^+$ for $C_{19}H_{20}NO_4SBr$ requires 437.02964; Anal Calc. for $C_{19}H_{20}NO_4SBr$: C, 52.06%; H, 4.60%; N, 3.20%; S, 7.32%. Found: C, 51.88%; H, 4.11%; N, 3.01%; S, 7.22%.

Compound 4k (R = I, *n* = 4)

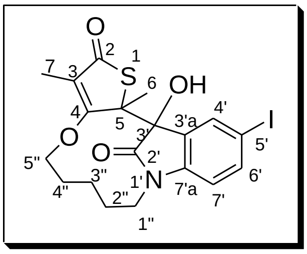

Light yellow solid (47.8 mg, 4%); mp 189–192 °C; R_f (EtOAc:Hex 2:3) 0.35; IRν_{max}(KBr)/cm^{-1} 3378 (OH), 1709 (C_2=O), 1601 ($C_{2'}$=O), 1482 (Ar C=C); δ_H (300MHz, DMSO-d$_6$) 7.90 (1H, d, *J* 1.8, H-4'), 7.41 (1H, dd, *J* 1.8 and 8.1, H-6'), 6.41 (1H, d, *J* 8.4, H-7'), 6.17 (1H, br. s, OH), 4.31 (1H, m, H-5"a/b), 3.96 (1H, m, H-1"a/b), 3.30 (1H, m, H-5"a/b), 3.04 (1H, dt, *J* 2.7 and 14.4, H-1"a/b), 1.82 (3H, s, H-7), 1.80-1.55 (4H, m, H-2" and H-4"), 1.48 (3H, s, H-6), 1.25 (2H, m, H-3"); δ_C (75MHz, DMSO-d$_6$) 195.0, 175.2, 174.8, 141.7, 138.2, 133.5, 130.6, 111.3, 109.2, 83.9, 78.6, 67.3, 65.7, 40.1, 27.9, 22.6, 19.6, 19.0, 9.3; HRMS(ESI) found *m/z* 486.02280 $[M+H]^+$ for $C_{19}H_{20}NO_4SI$ requires 485.01577; Anal Calc. for $C_{19}H_{20}NO_4SI$: C, 47.02%; H, 4.15%; N, 2.89%; S, 6.61%. Found: C, 46.98%; H, 3.97%; N, 2.57%; S, 6.22%.

Compound 4l (R = Cl , *n* = 5)

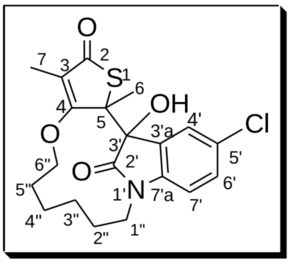

Yellow crystalline solid (65.5 mg, 6%); mp 214–215 °C; R_f (EtOAc:Hex 1:1) 0.48; IRν_{max}(KBr)/cm^{-1} 3367 (OH), 1699 (C_2=O), 1611 ($C_{2'}$=O), 1478 (Ar C=C); δ_H (400MHz, $CDCl_3$) 7.87 (1H, d, *J* 2.4, H-4'), 7.28 (1H, dd, *J* 2.4 and 8.4, H-6'), 6.78 (1H, d, *J* 8.4, H-7'), 4.29–4.14 (3H, m, H-1"a/b and H-6"), 3.30 (1H, br. s, OH), 3.26 (1H, m, H-1"a/b), 2.05 (3H, s, H-7), 1.98-1.84 (3H, m, H-2"a/b and H-5"), 1.74 (3H, s, H-6), 1.64-1.30 (5H, m, H-2"a/b, H-3" and H-4"); δ_C (100MHz, $CDCl_3$) 194.8, 175.9, 175.0, 142.6, 130.5, 128.9, 125.7, 125.0, 112.0, 109.3, 77.4, 72.6, 65.2, 39.5, 26.0, 24.1, 23.7, 22.6, 21.5, 9.8; HRMS(EI) found *m/z* 407.09528 for $C_{20}H_{22}O_4ClNS$ requires 407.09581; Anal Calc. for $C_{20}H_{22}O_4ClNS$: C, 58.89%; H, 5.44%; N, 3.43%; S, 7.86%. Found: C, 58.85%; H, 5.42%; N, 3.38%; S, 7.89%

Compound 4m (R = F, *n* =5)

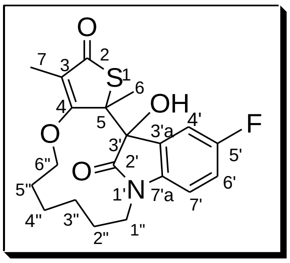

Light yellow crystalline solid (74.5 mg, 7%); mp 212–213 °C; R_f (EtOAc:Hex 1:1) 0.45; IRν_{max}(KBr)/cm^{-1} 3358 (OH), 1699 (C_2=O), 1614 ($C_{2'}$=O), 1491 (Ar C=C); δ_H (400MHz, $CDCl_3$) 7.40 (1H, dd, *J* 2.8 and 8.8, H-4'), 6.78 (1H, ddd, *J* 2.8, 8.4 and 8.8, H-6'), 6.57 (1H, dd, *J* 4.0 and 8.4, H-7'), 4.08–3.97 (3H, m, H-1"a/b and H-6"), 3.06 (1H, m, H-1"a/b), 2.52 (1H, br. s, OH), 1.89 (3H, s, H-7), 1.78-1.66 (3H, m, H-2"a/b and H-5"), 1.54 (3H, s, H-6), 1.43–1.24 (5H, m, H-2"a/b, H-3" and H-4"); δ_C (100MHz, $CDCl_3$) 195.5, 176.3, 175.0, 159.4, 157.0, 139.9, 129.8, 116.0, 113.2, 111.2, 108.4, 76.5, 72.2, 65.4, 39.0, 25.8, 23.7, 23.5, 22.4, 21.6, 9.7; HRMS(EI) found *m/z* 391.12484 for $C_{20}H_{22}O_4FNS$ requires 391.12536; Anal Calc. for $C_{20}H_{22}O_4FNS$: C, 61.36%; H, 5.66%; N, 3.58%; S, 8.19%. Found: C, 60.92%; H, 5.74%; N, 3.24%; S, 7.98%.

Compound 4n (R = Br, *n* = 5)

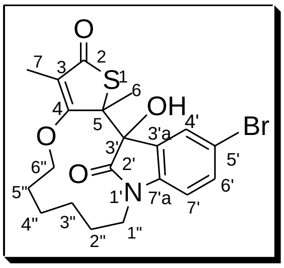

Yellow crystalline solid (68.9 mg, 6%); mp 211 °C; R_f (EtOAc:Hex 1:1) 0.55; IRν_{max}(KBr)/cm^{-1} 3370 (OH), 1698 (C_2=O), 1609 ($C_{2'}$=O), 1478 (Ar C=C); δ_H (400MHz, $CDCl_3$) 8.00 (1H, d, *J* 2.0, H-4'), 7.44 (1H, dd, *J* 2.0 and 8.4, H-6'), 6.73 (1H, br. d, *J* 8.4, H-7'), 4.29–4.15 (3H, m, H-1"a/b and H-6"), 3.27 (2H, m, H-1"a/b and OH), 2.05 (3H, s, H-7), 1.96–1.85 (3H, m, H-2"a/b and H-5"), 1.75 (3H, s, H-6), 1.63-1.31 (5H, m, H-2"a/b, H-3" and H-4"); δ_C (100MHz, $CDCl_3$) 194.7, 175.8, 174.8, 143.1, 133.4, 129.2, 128.4, 115.3, 112.0, 109.8, 76.7, 72.6, 65.2, 39.4, 26.0, 24.1, 23.7, 22.6, 21.5, 9.9; HRMS(EI) found *m/z* 451.04475 for $C_{20}H_{22}O_4FNS$ requires 451.04529; Anal Calc. for $C_{20}H_{22}O_4BrNS$: C, 53.10%; H, 4.90%; N, 3.10%; S, 7.09%. Found: C, 53.19%; H, 4.89%; N, 2.94%; S, 6.97%.

Compound 4o (R = I, *n* = 5)

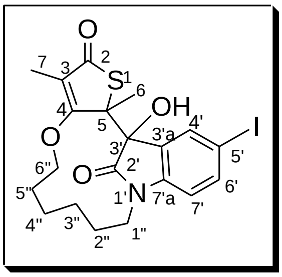

Yellow crystalline solid (90.8 mg, 7%); mp 214–215°C; R_f (EtOAc:Hex 1:1) 0.45; IRν_{max}(KBr)/cm^{-1} 3365 (OH), 1697 (C_2=O), 1604 ($C_{2'}$=O), 1481 (Ar C=C); δ_H (400MHz, $CDCl_3$) 7.99 (1H, d, *J* 1.6, H-4’), 7.46 (1H, dd, *J* 2.0 and 8.0, H-6’), 6.49 (1H, d, *J* 8.0, H-7’), 4.15-4.02 (3H, m, H-1”a/b and H-6”), 3.10 (1H, m, H-1”a/b), 2.40 (1H, br. s, OH), 1.94 (3H, s, H-7), 1.84–1.70 (3H, m, H-2”a/b and H-5”), 1.61 (3H, s, H-6), 1.51–1.15 (5H, m, H-2”a/b, H-3” and H-4”); δ_C (100MHz, $CDCl_3$) 195.1, 176.0, 174.4, 143.5, 138.5, 133.5, 130.2, 111.2, 109.8, 83.9, 76.2, 72.1, 65.2, 38.8, 25.6, 23.7, 23.3, 22.2, 21.3, 9.5; HRMS(EI) found *m/z* 499.03088 for $C_{20}H_{22}O_4INS$ requires 499.03142; Anal Calc. for $C_{20}H_{22}O_4INS$: C, 48.10%; H, 4.44%; N, 2.80%; S, 6.42%. Found: C, 48.14%; H, 4.50%; N, 2.69%; S, 6.23%.

^{1}H NMR and ^{13}C NMR Spectra

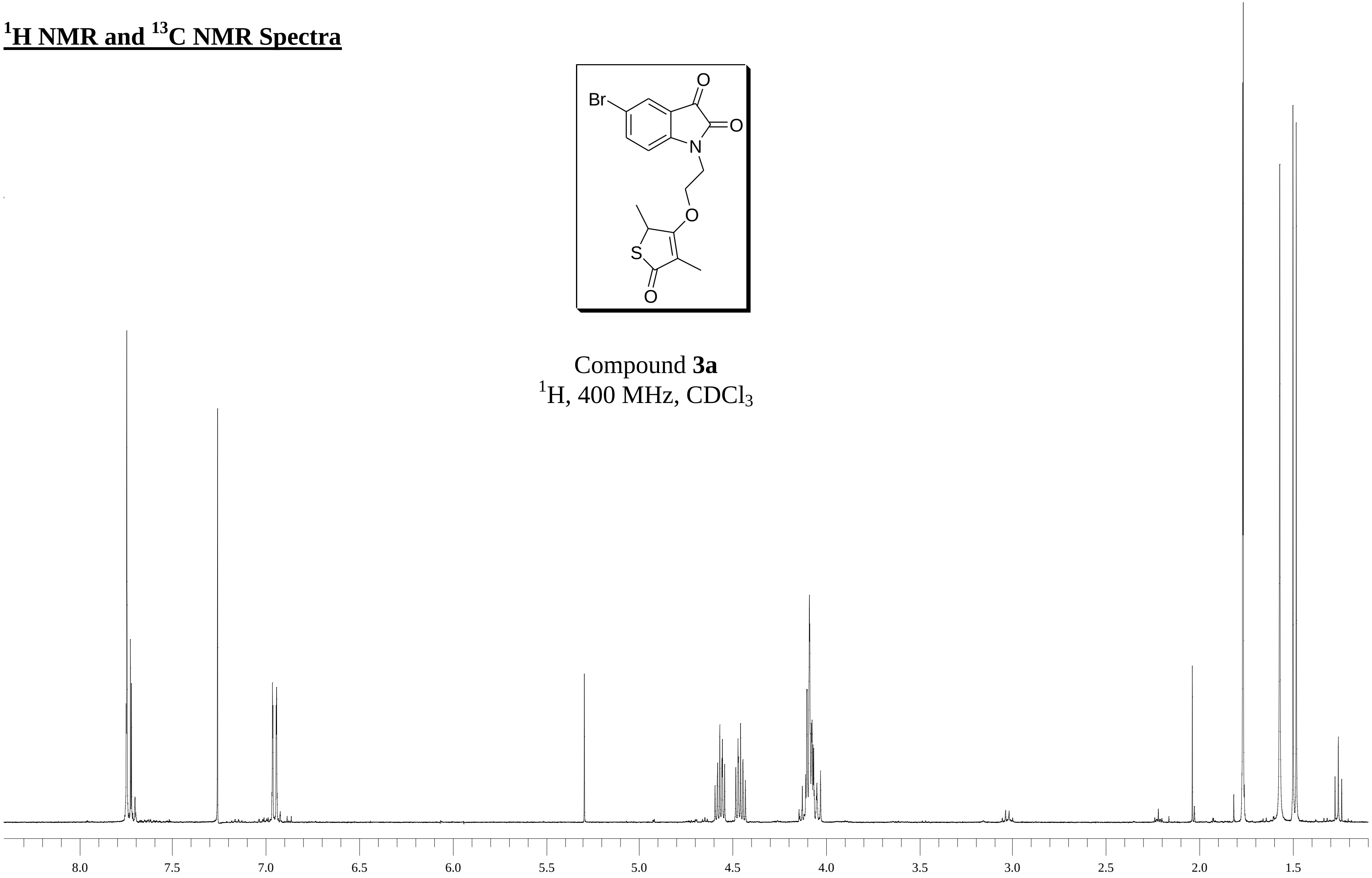

Compound **3a**

^{1}H, 400 MHz, $CDCl_3$

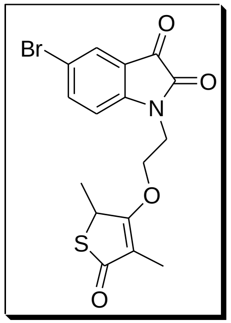

Compound **3a**
^{13}C, 100 MHz, $CDCl_3$

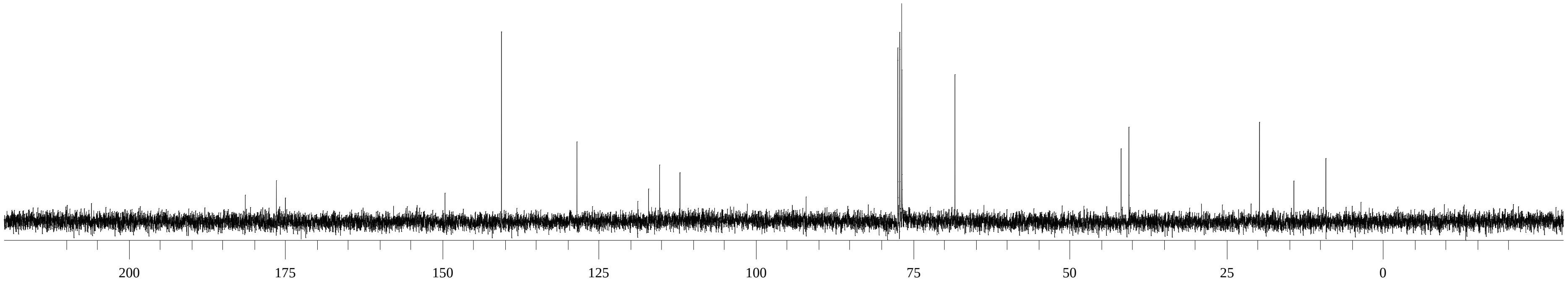

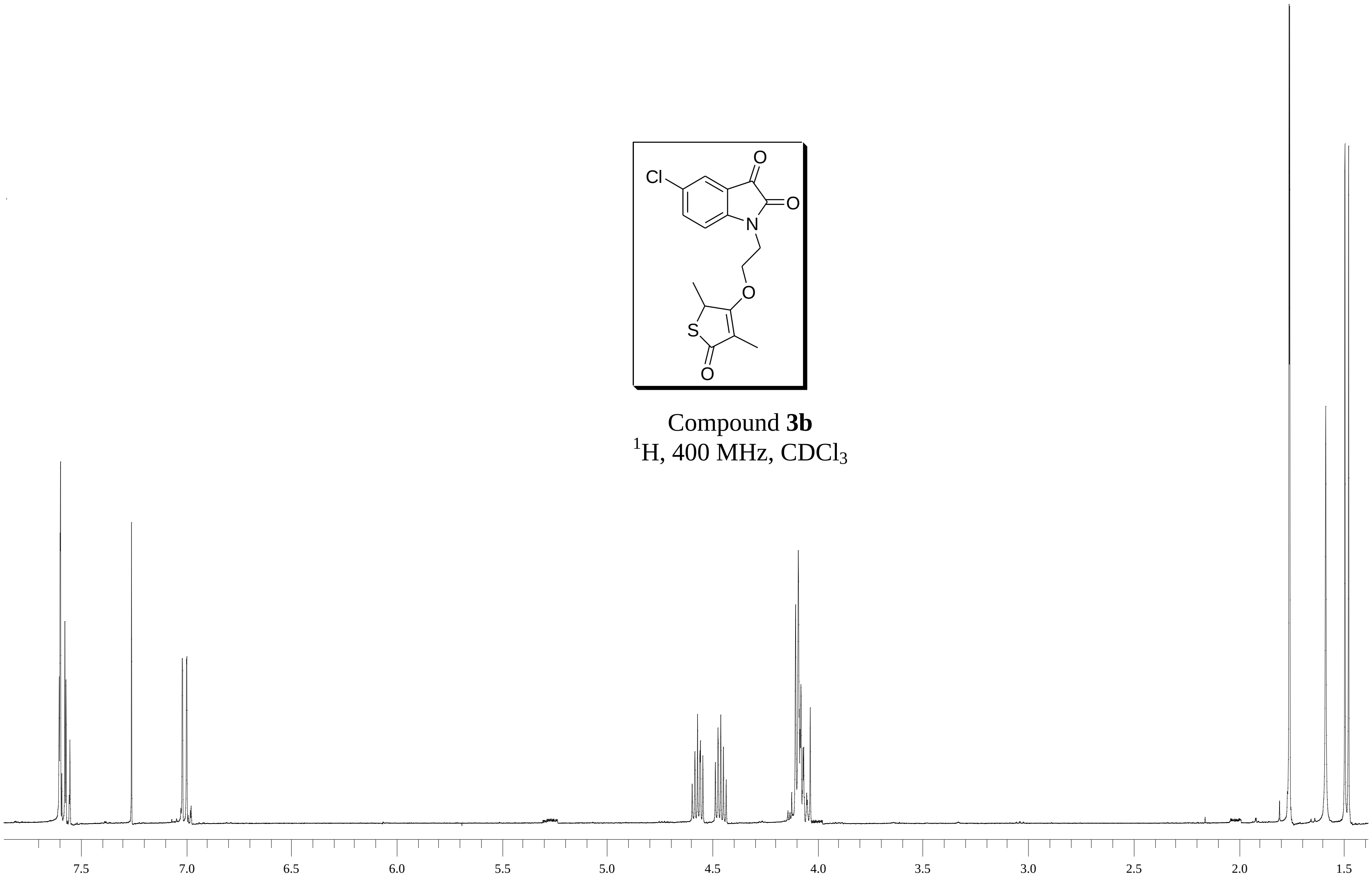

Compound **3b**
^{1}H, 400 MHz, $CDCl_3$

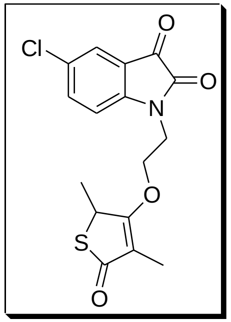

Compound **3b**

^{13}C, 100 MHz, $CDCl_3$

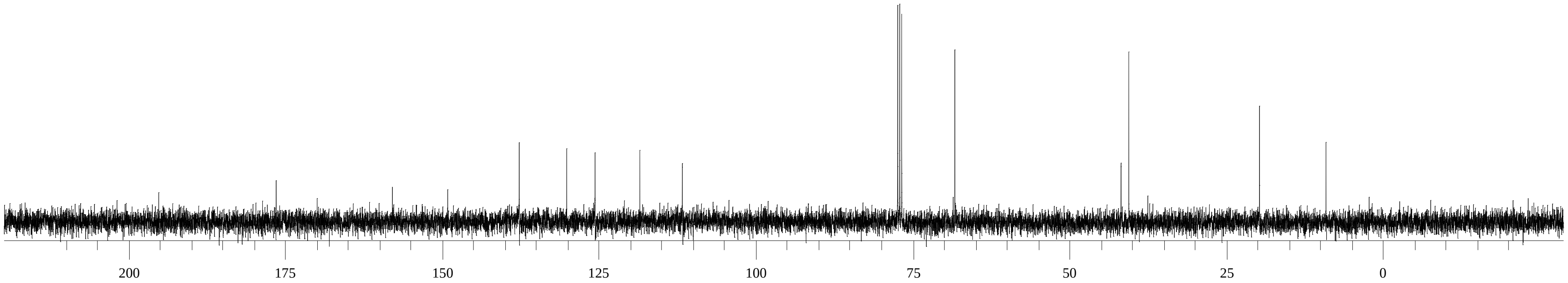

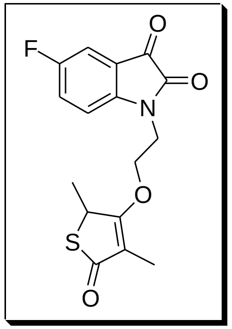

Compound **3c**
^{1}H, 400 MHz, $CDCl_3$

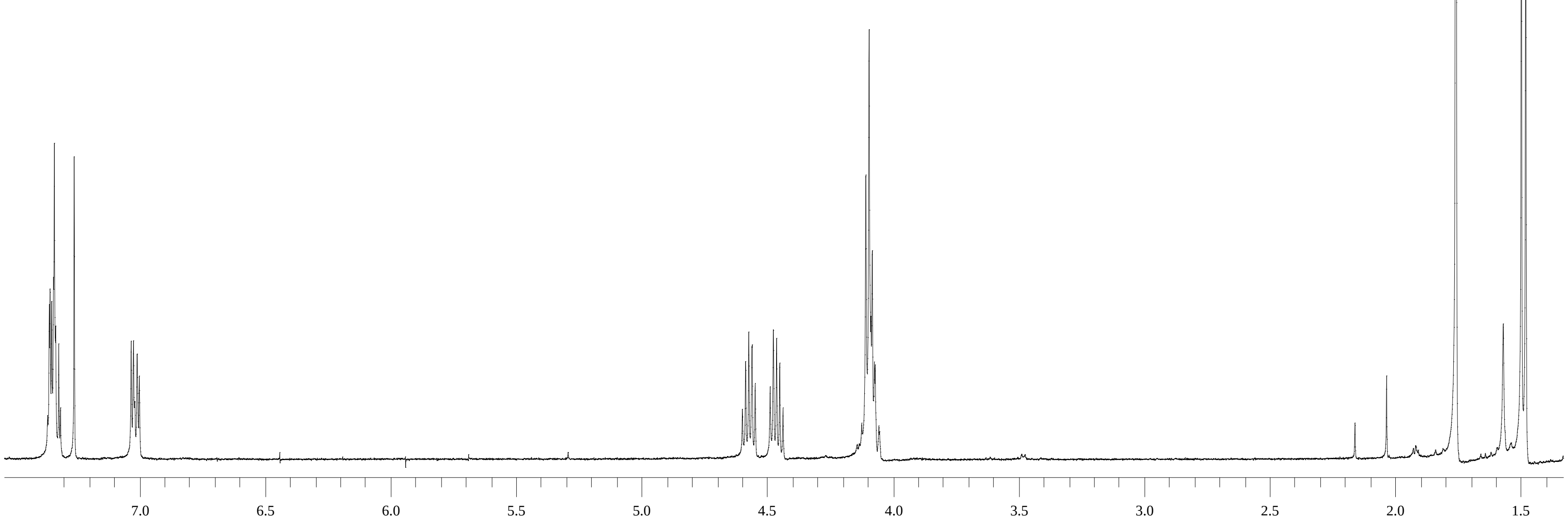

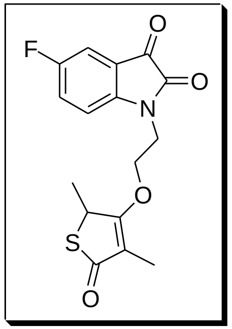

Compound **3c**

^{13}C, 100 MHz, $CDCl_3$

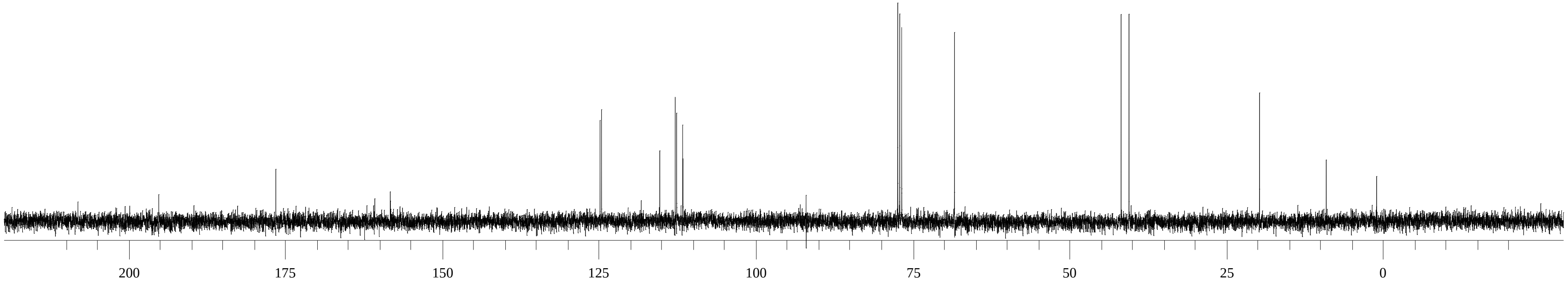

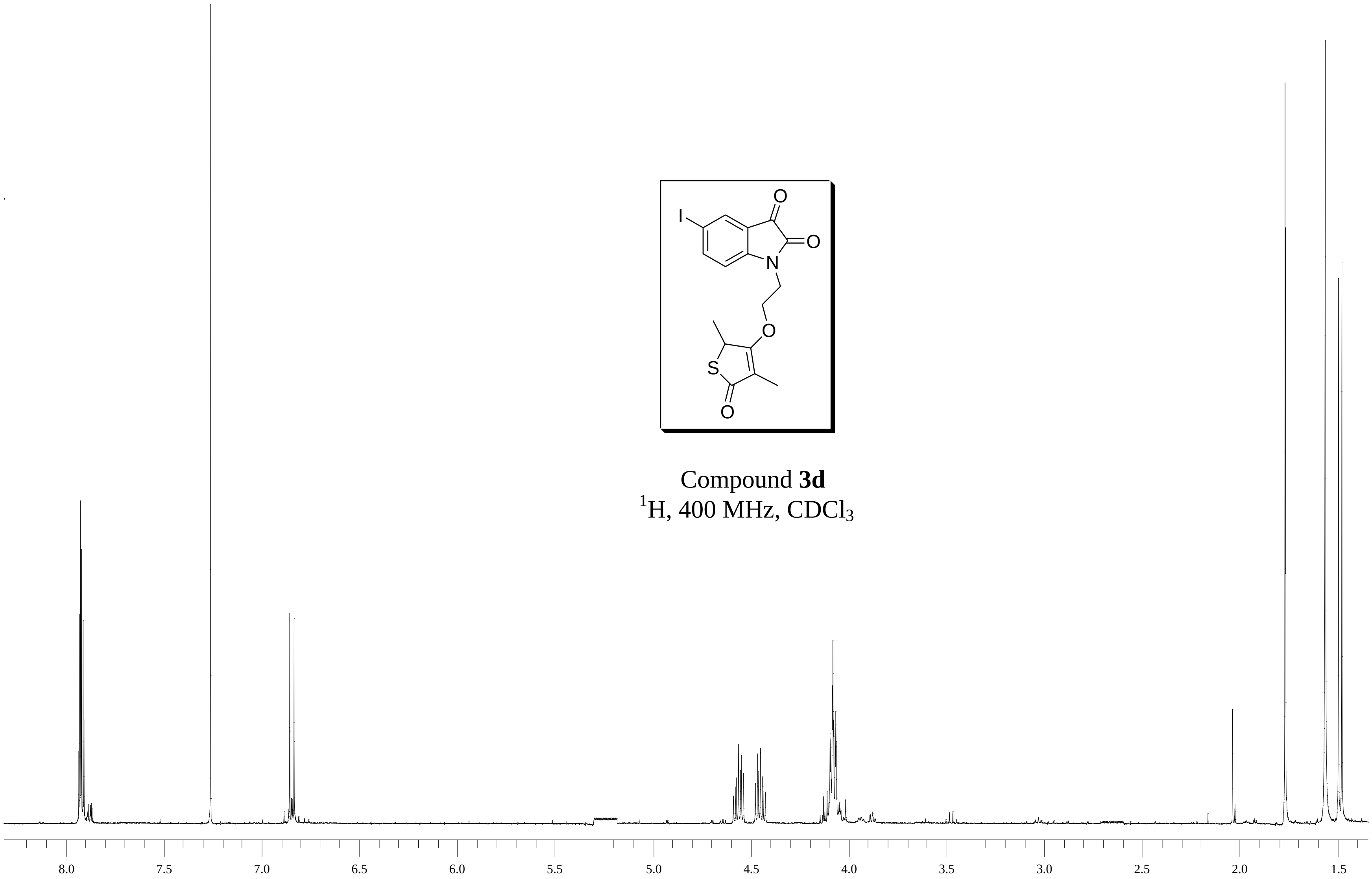

Compound **3d**
^{1}H, 400 MHz, $CDCl_3$

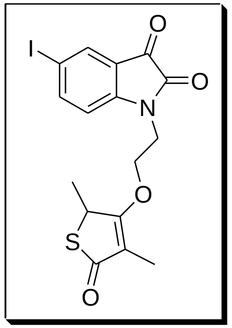

Compound **3d**

^{13}C, 100 MHz, $CDCl_3$

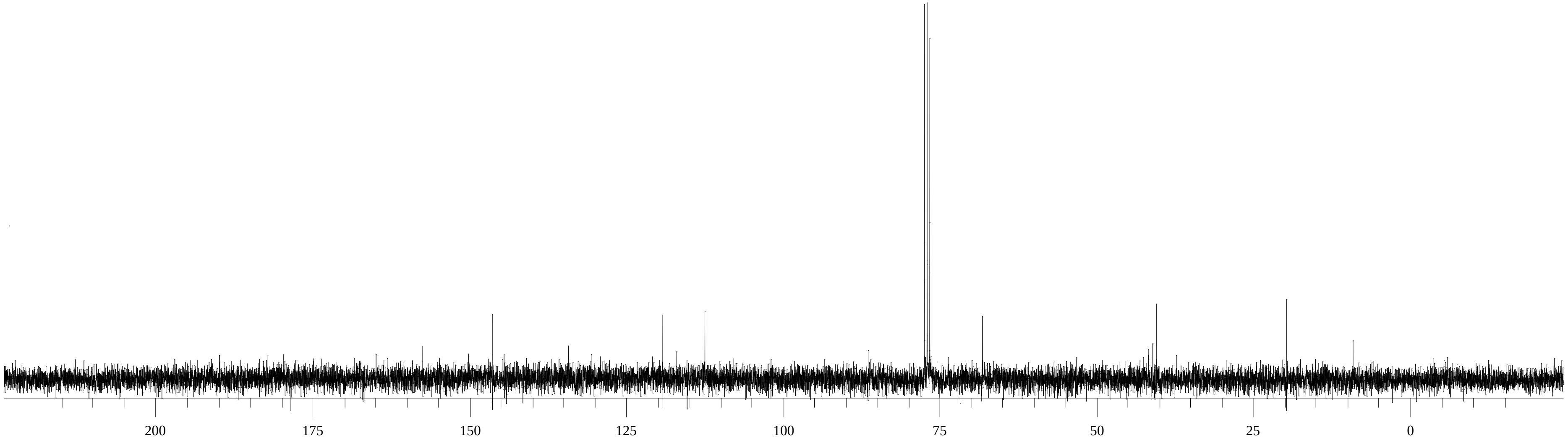

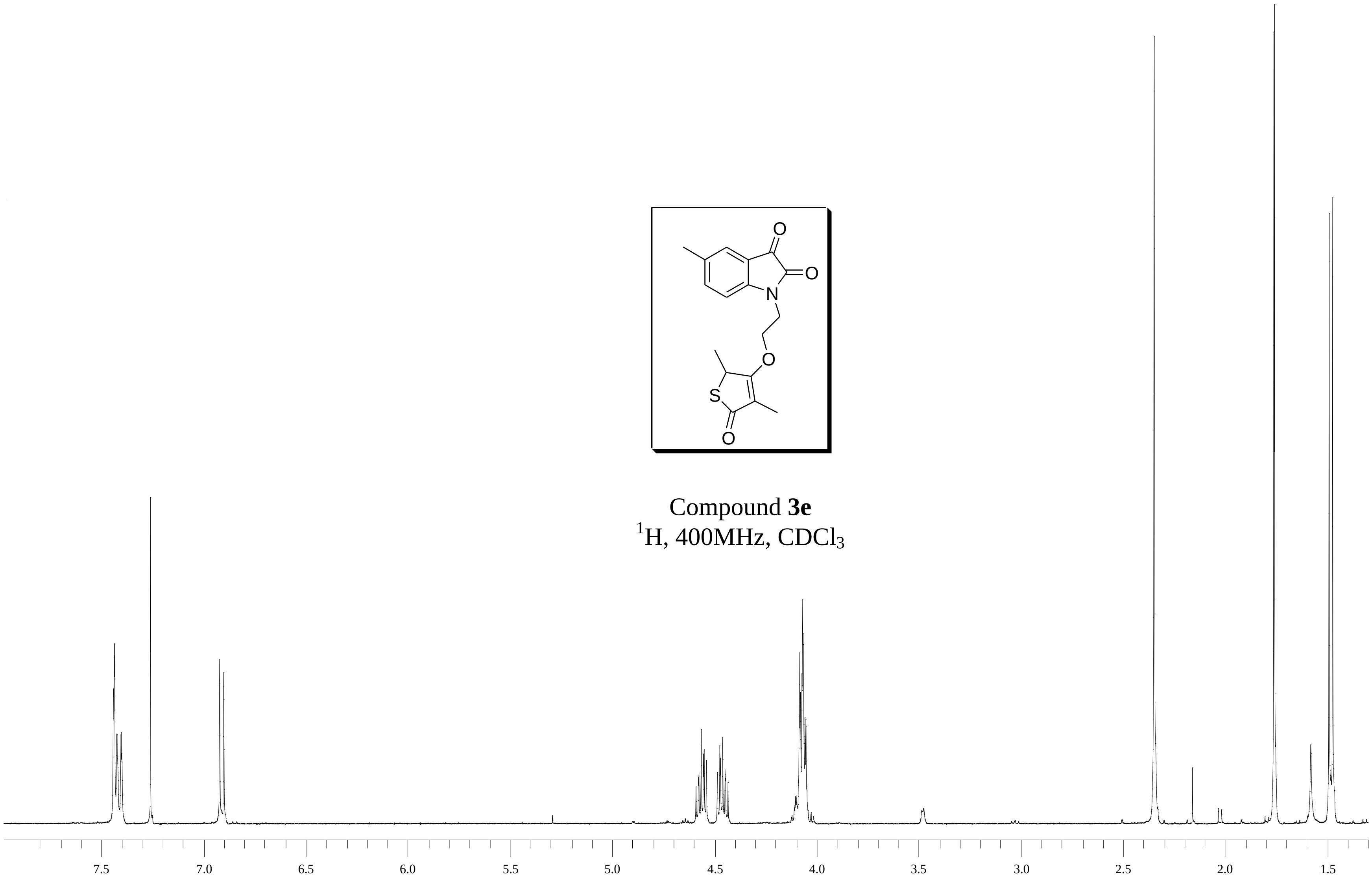

Compound **3e**
^{1}H, 400MHz, $CDCl_3$

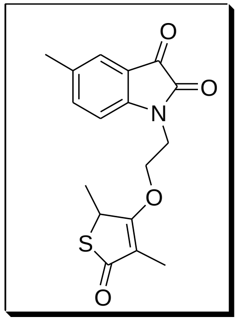

Compound **3e**
^{13}C, 100 MHz, $CDCl_3$

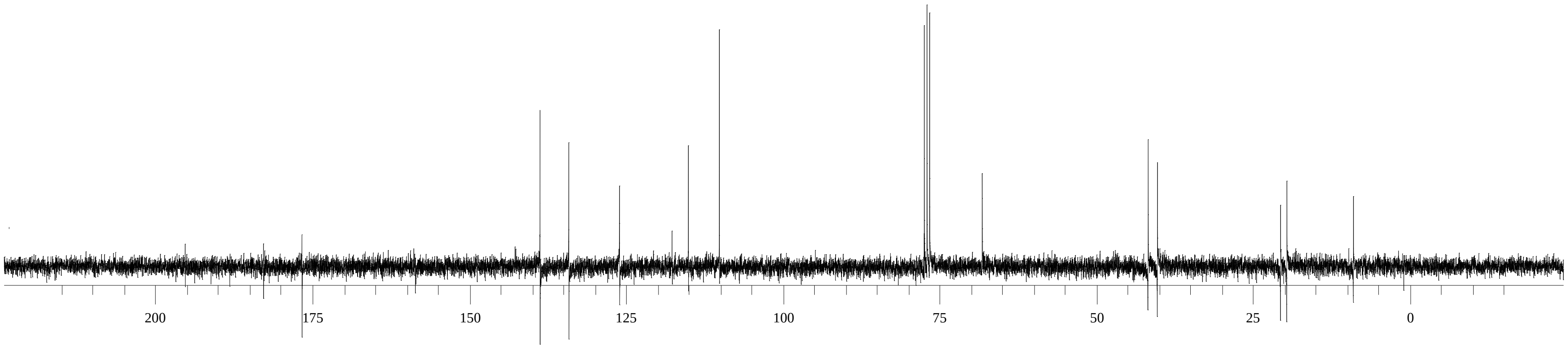

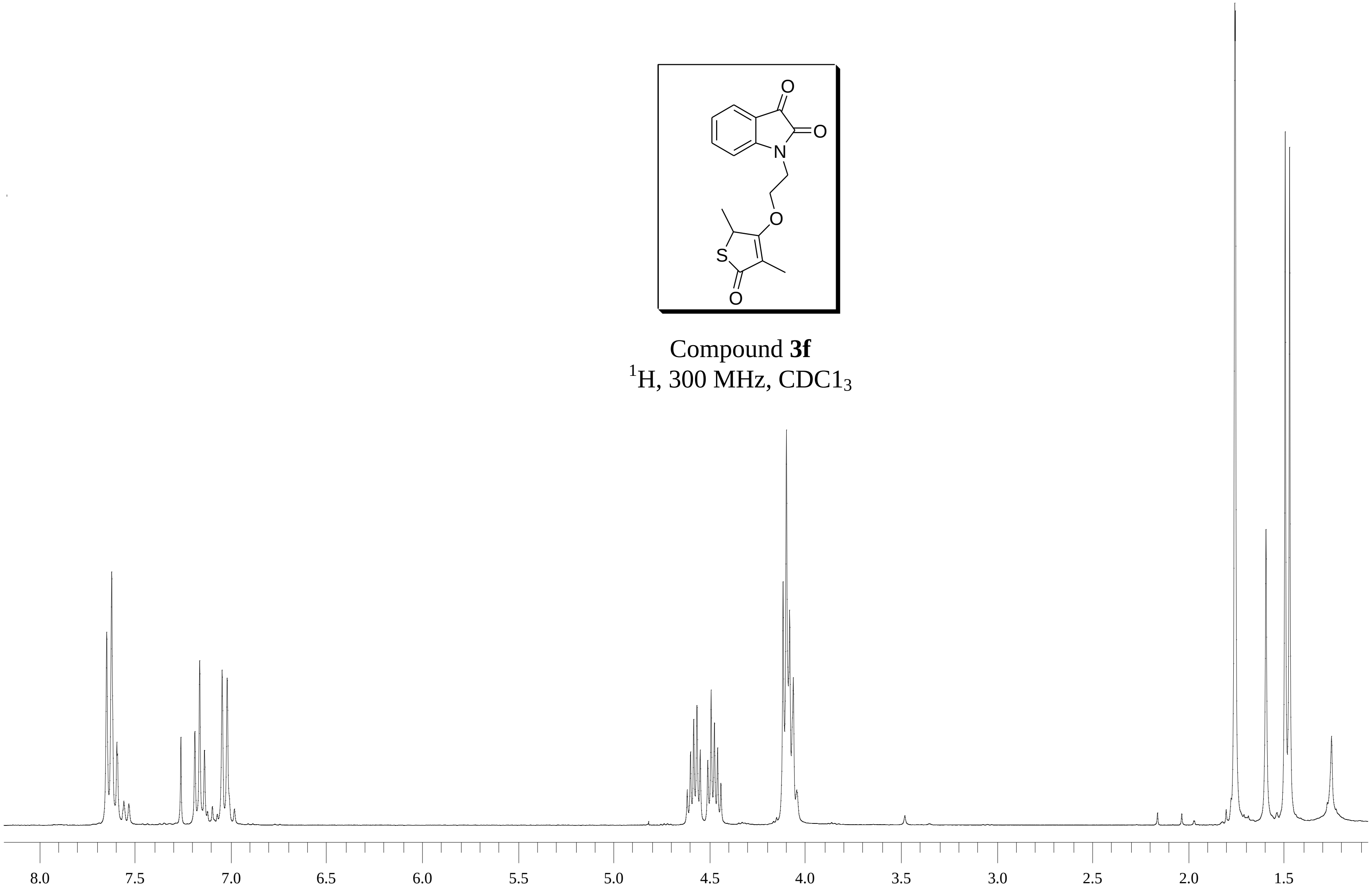

Compound **3f**
^{1}H, 300 MHz, CDC1$_3$

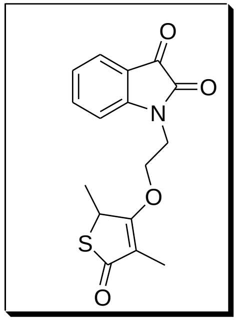

Compound **3f**
^{13}C, 100 MHz, $CDCl_3$

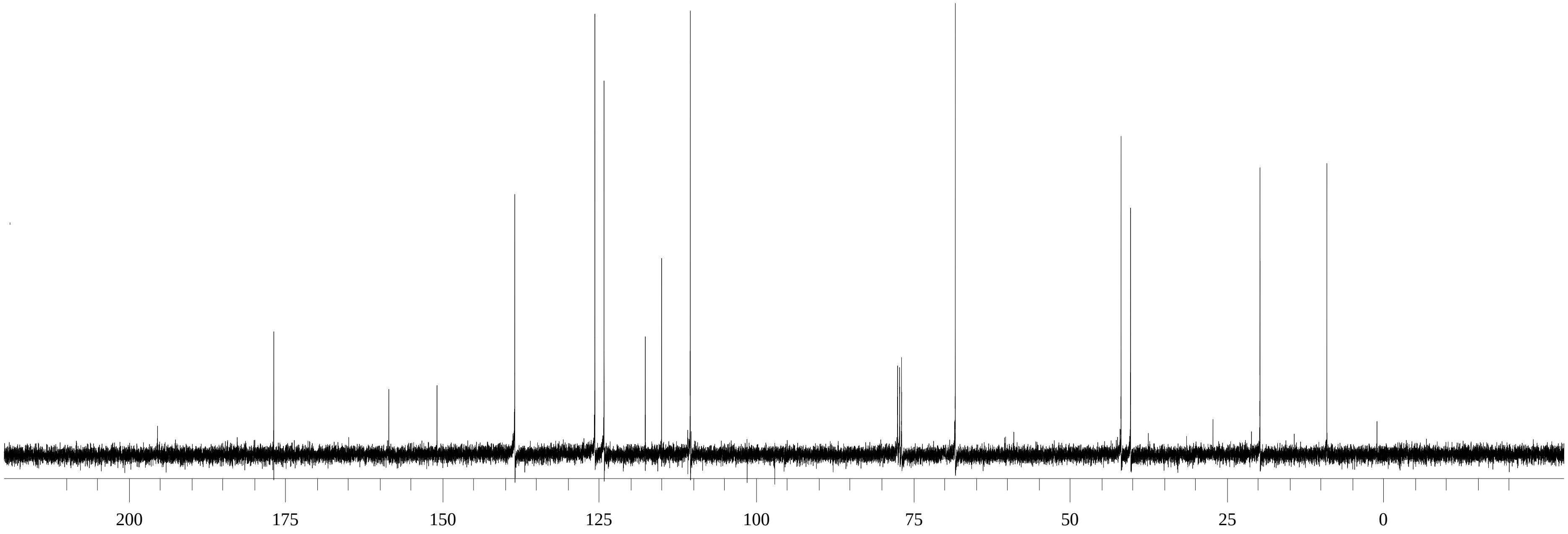

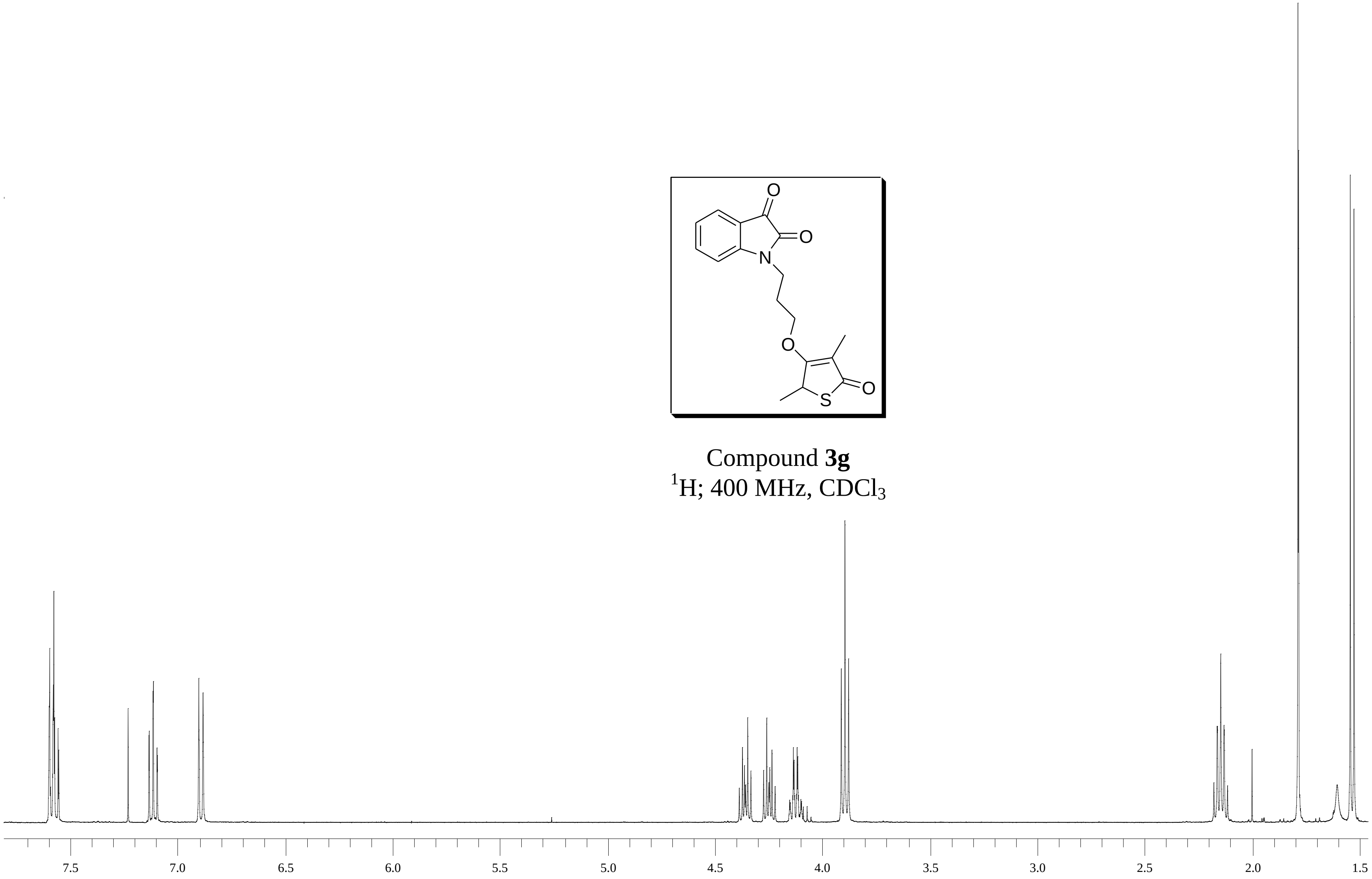

Compound **3g**
^{1}H; 400 MHz, $CDCl_3$

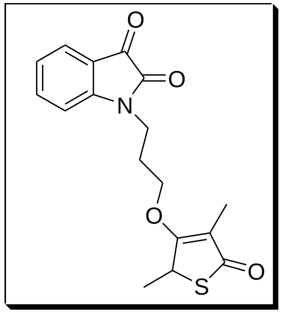

Compound **3g**
^{13}C; 100 MHz, $CDCl_3$

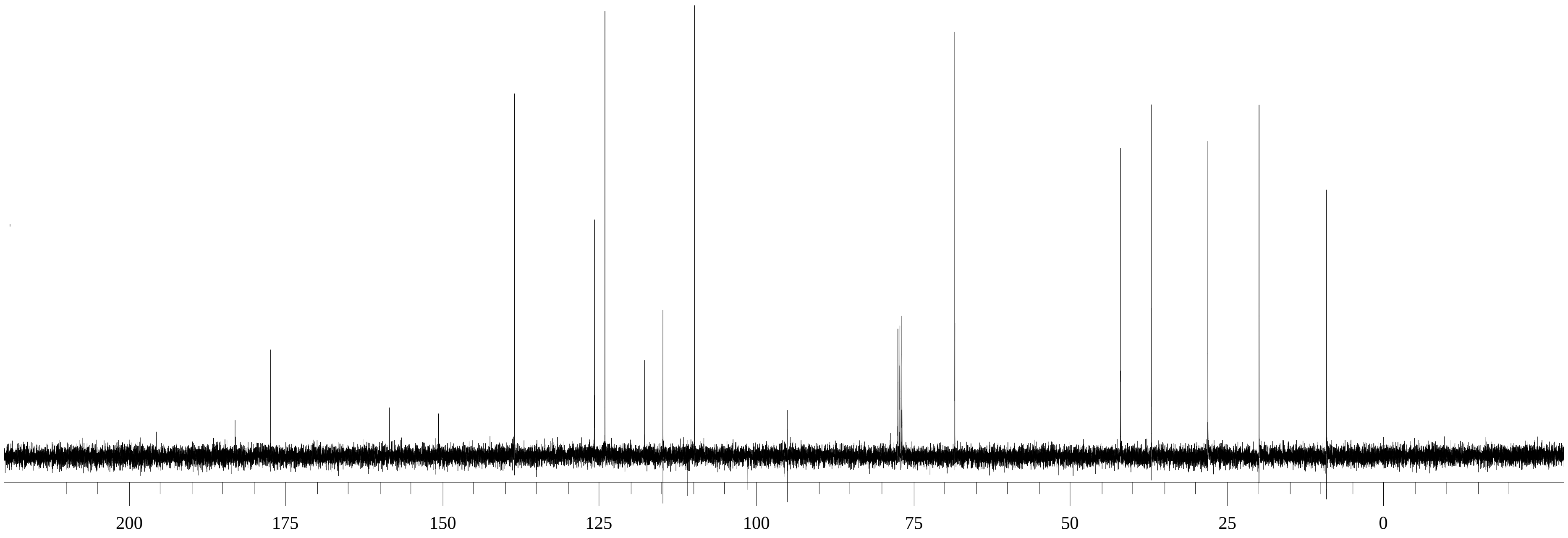

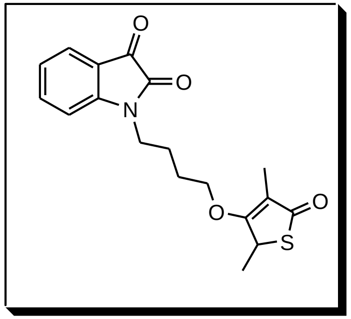

Compound **3h**

^{1}H, 300 MHz, $CDCl_3$

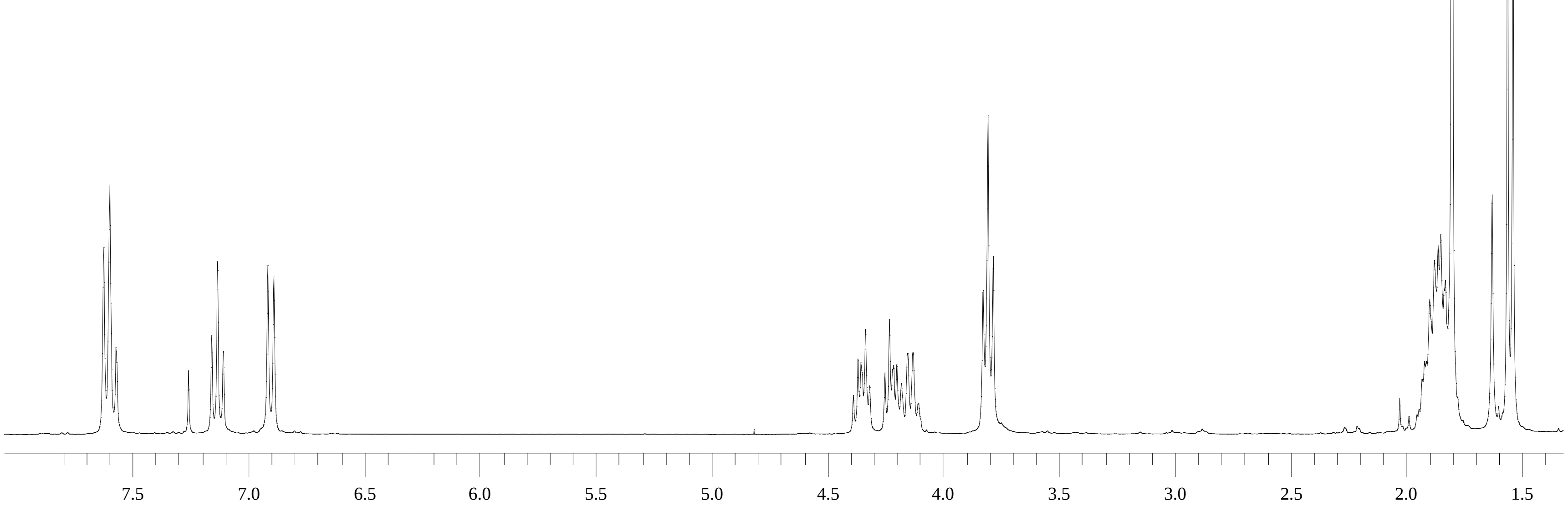

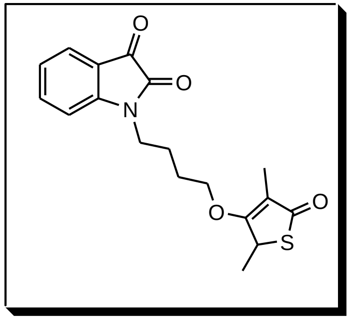

Compound **3h**

^{13}C, 75 MHz, $CDCl_3$

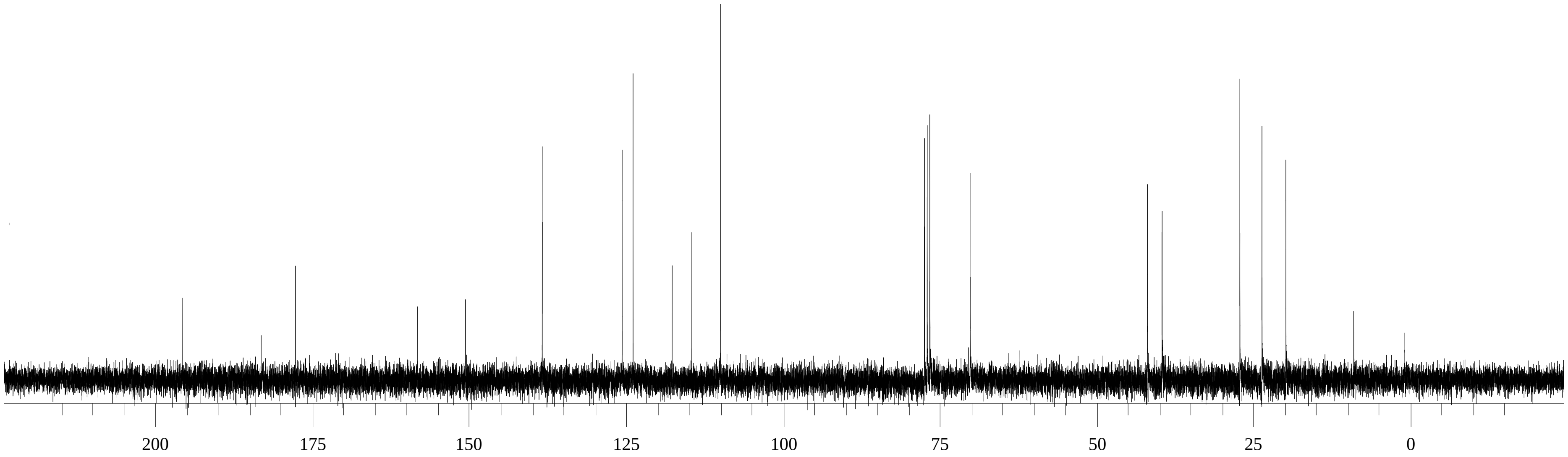

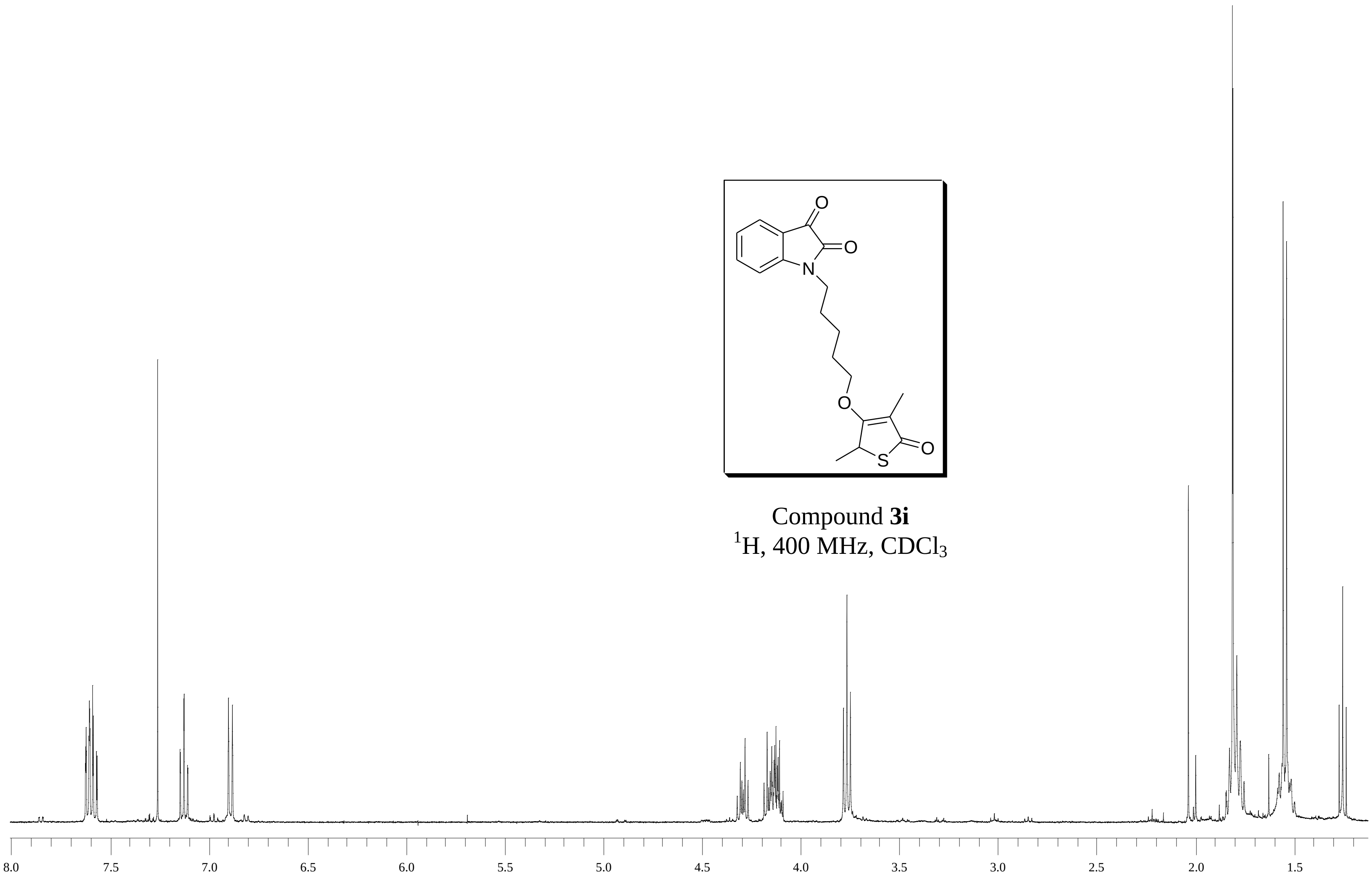

Compound **3i**
^{1}H, 400 MHz, $CDCl_3$

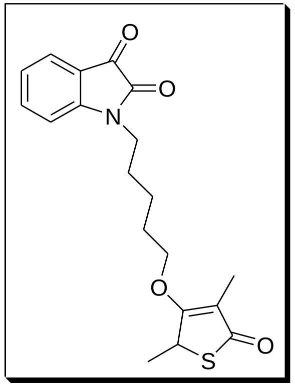

Compound **3i**
^{13}C, 100 MHz, $CDCl_3$

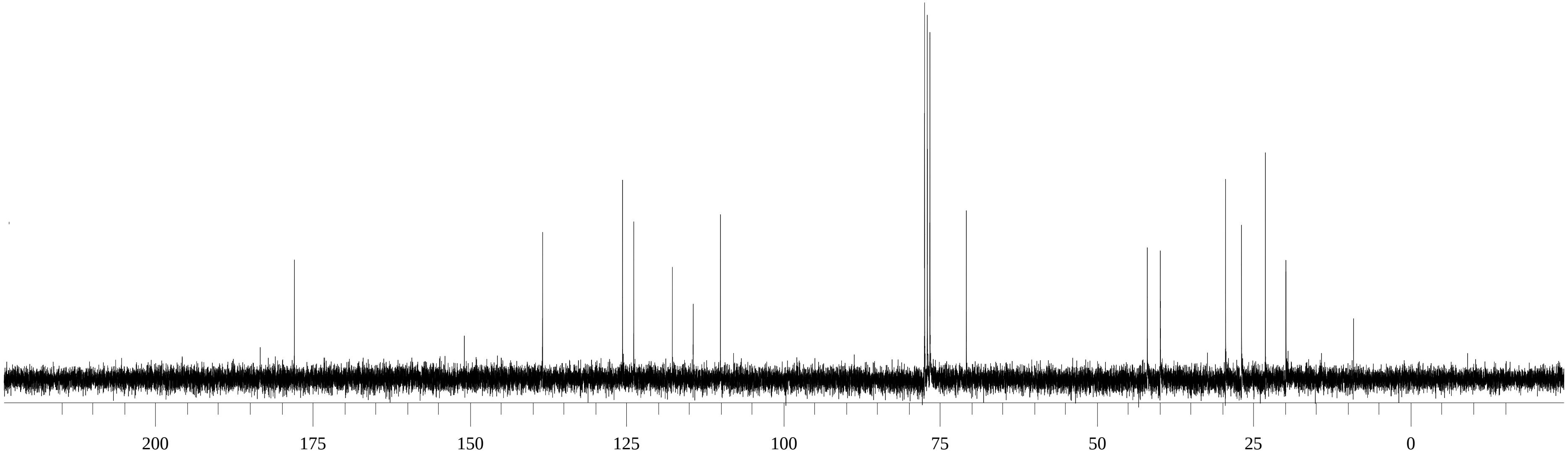

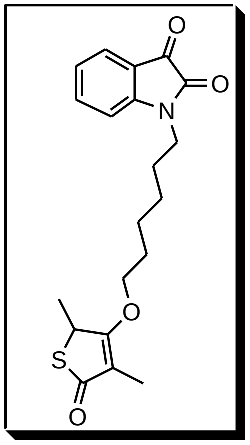

Compound **3j**
^{1}H, 300 MHz, $CDCl_3$

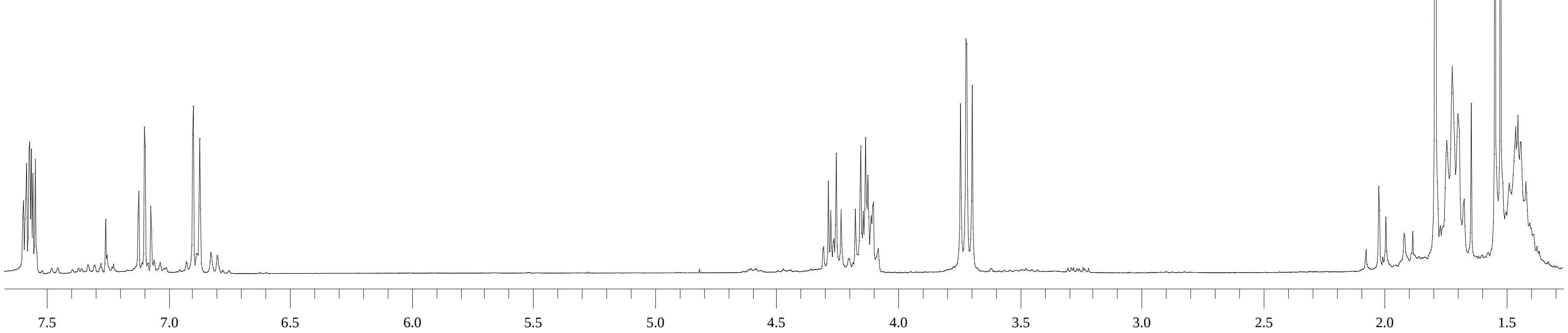

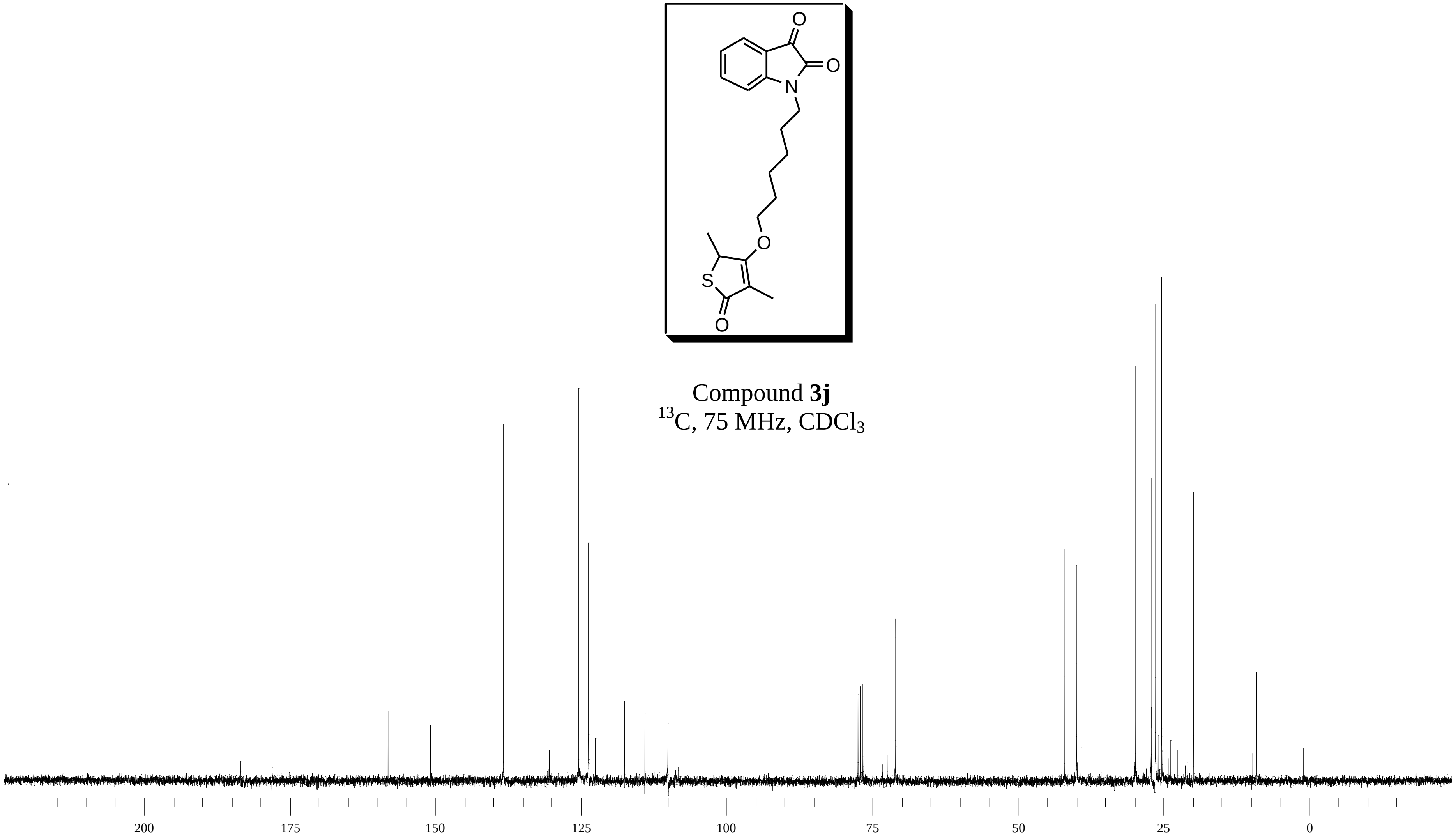

Compound **3j**
^{13}C, 75 MHz, $CDCl_3$

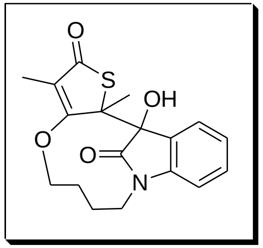

Compound **4a**
^{1}H, 400 MHz, $CDCl_3$

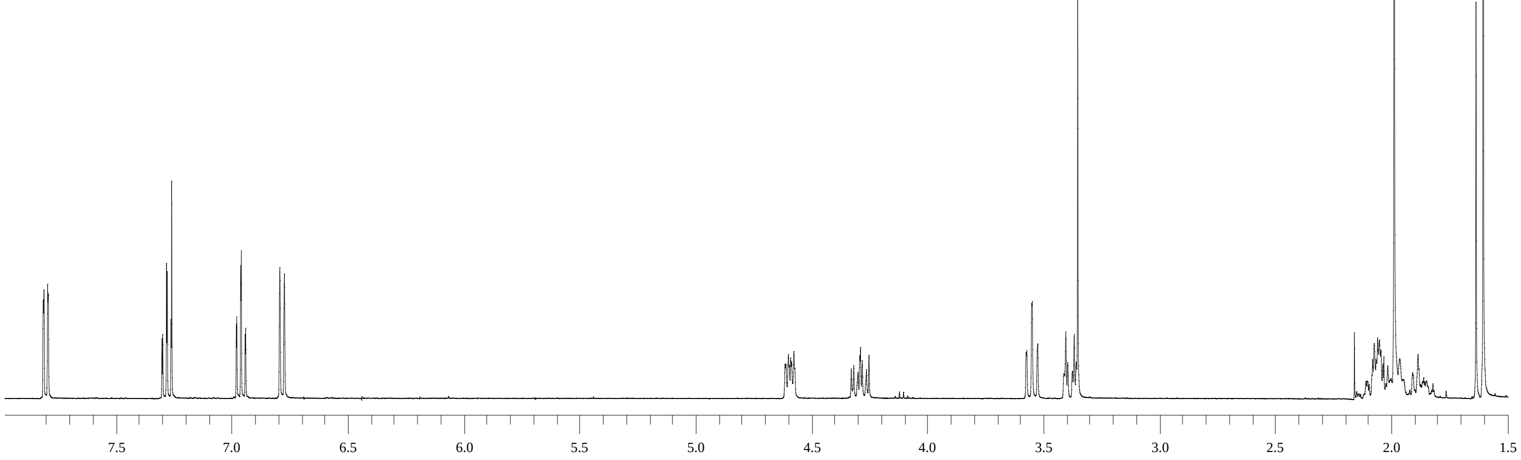

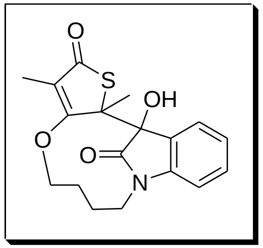

Compound **4a**

^{13}C, 100 MHz, $CDCl_3$

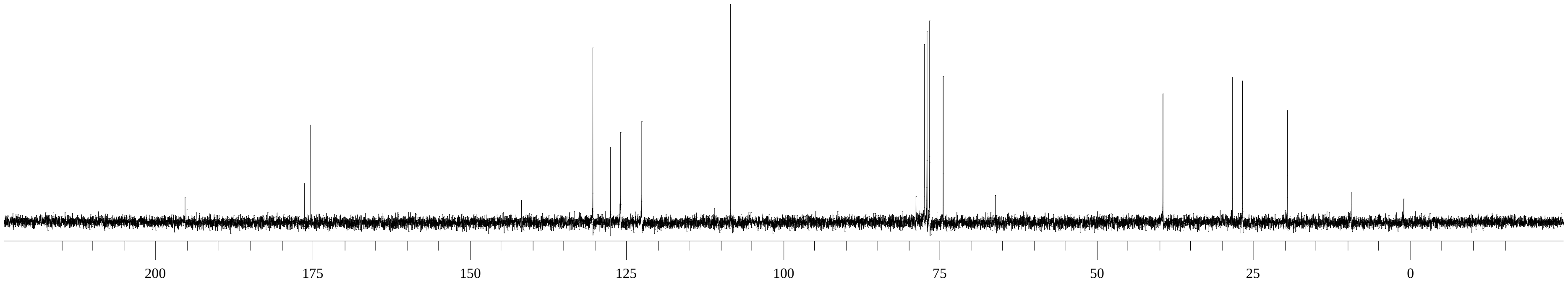

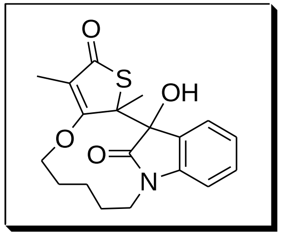

Compound **4b**
^{1}H, 400 MHz, $CDCl_3$

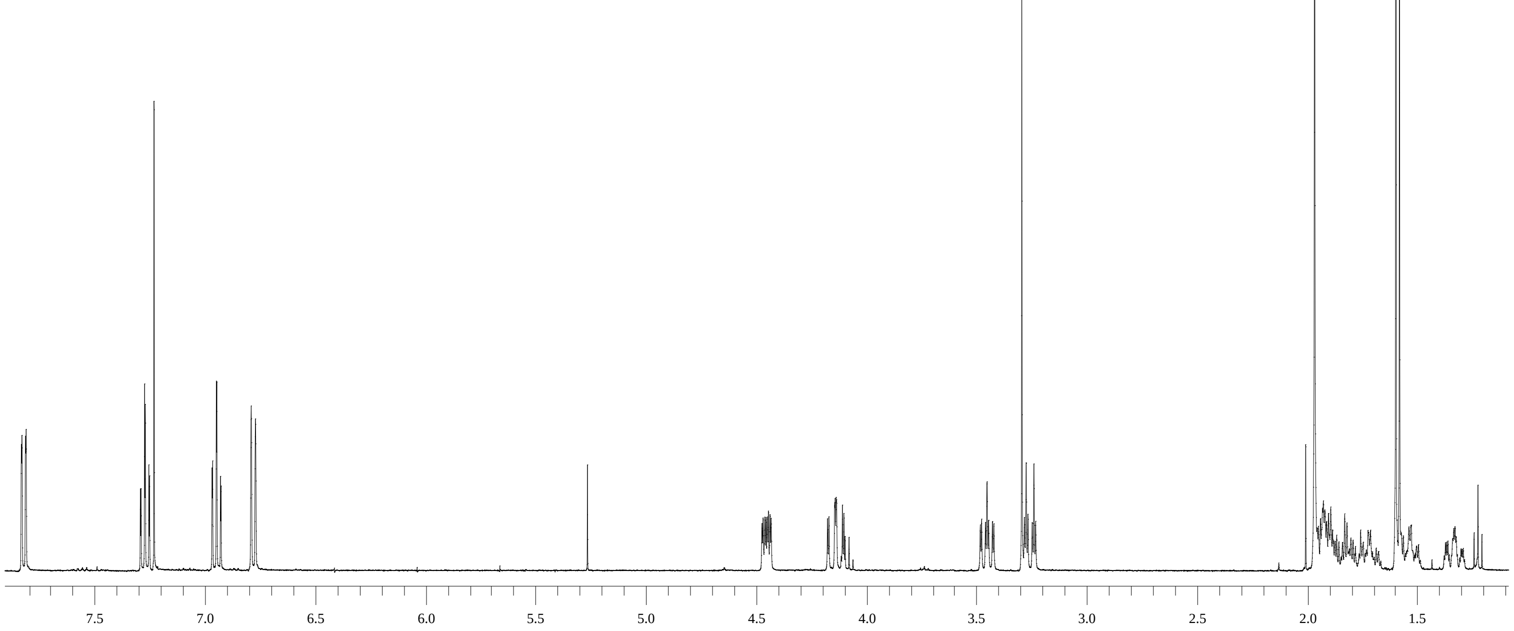

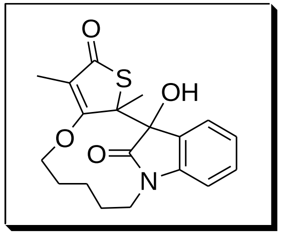

Compound **4b**
^{13}C, 100 MHz, $CDCl_3$

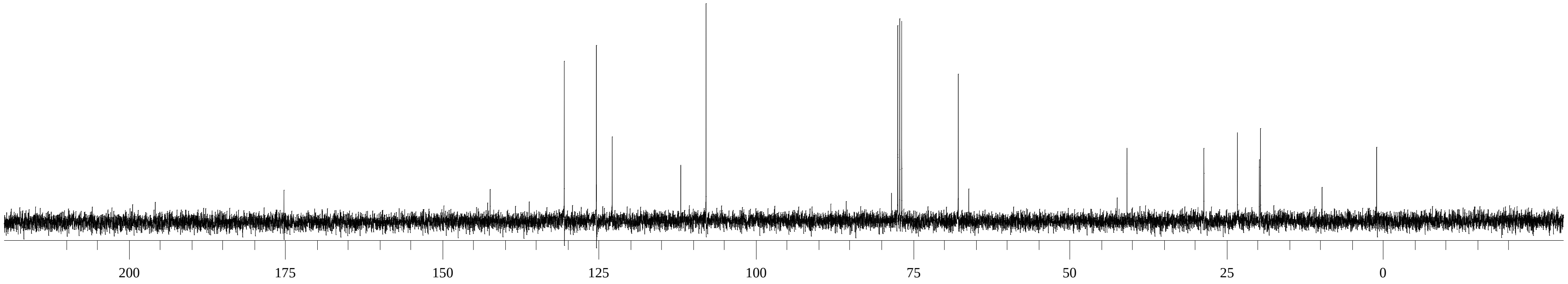

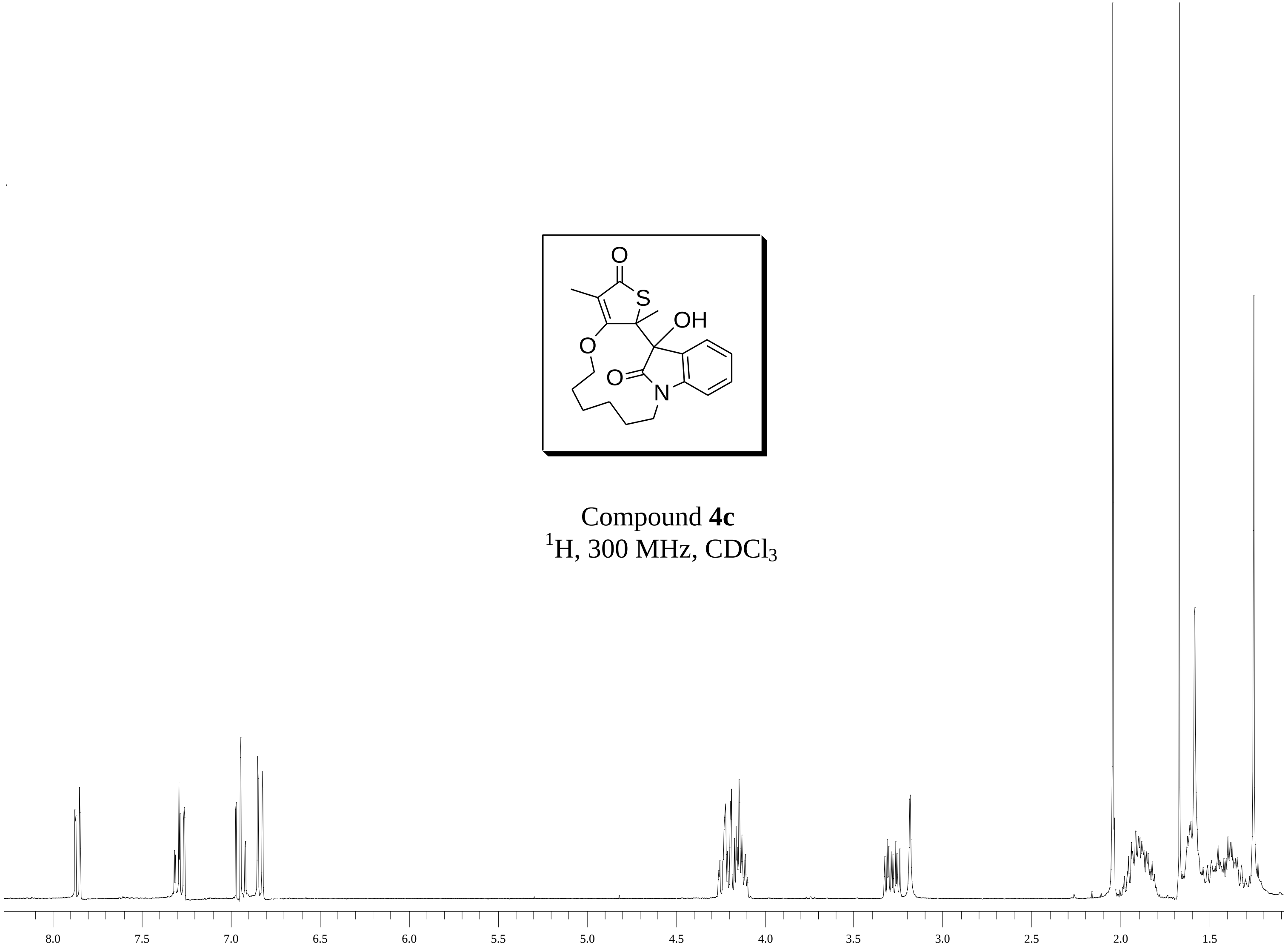

Compound **4c**
^{1}H, 300 MHz, $CDCl_3$

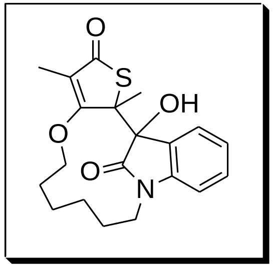

Compound **4c**
^{13}C, 75 MHz, $CDCl_3$

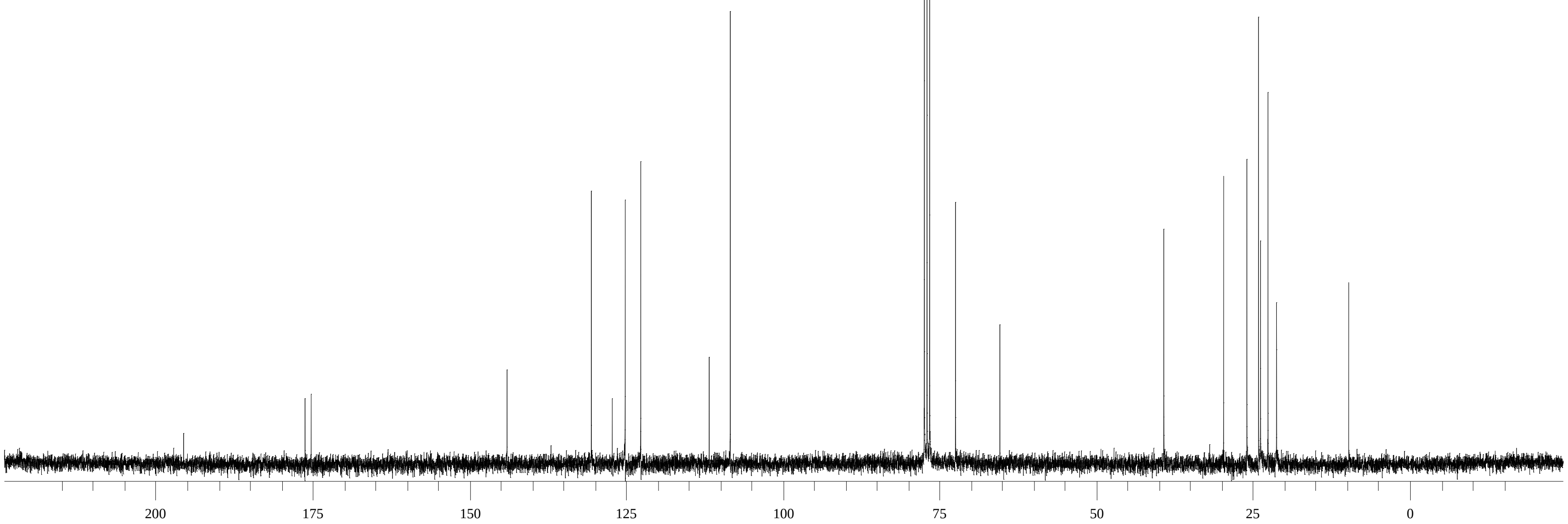

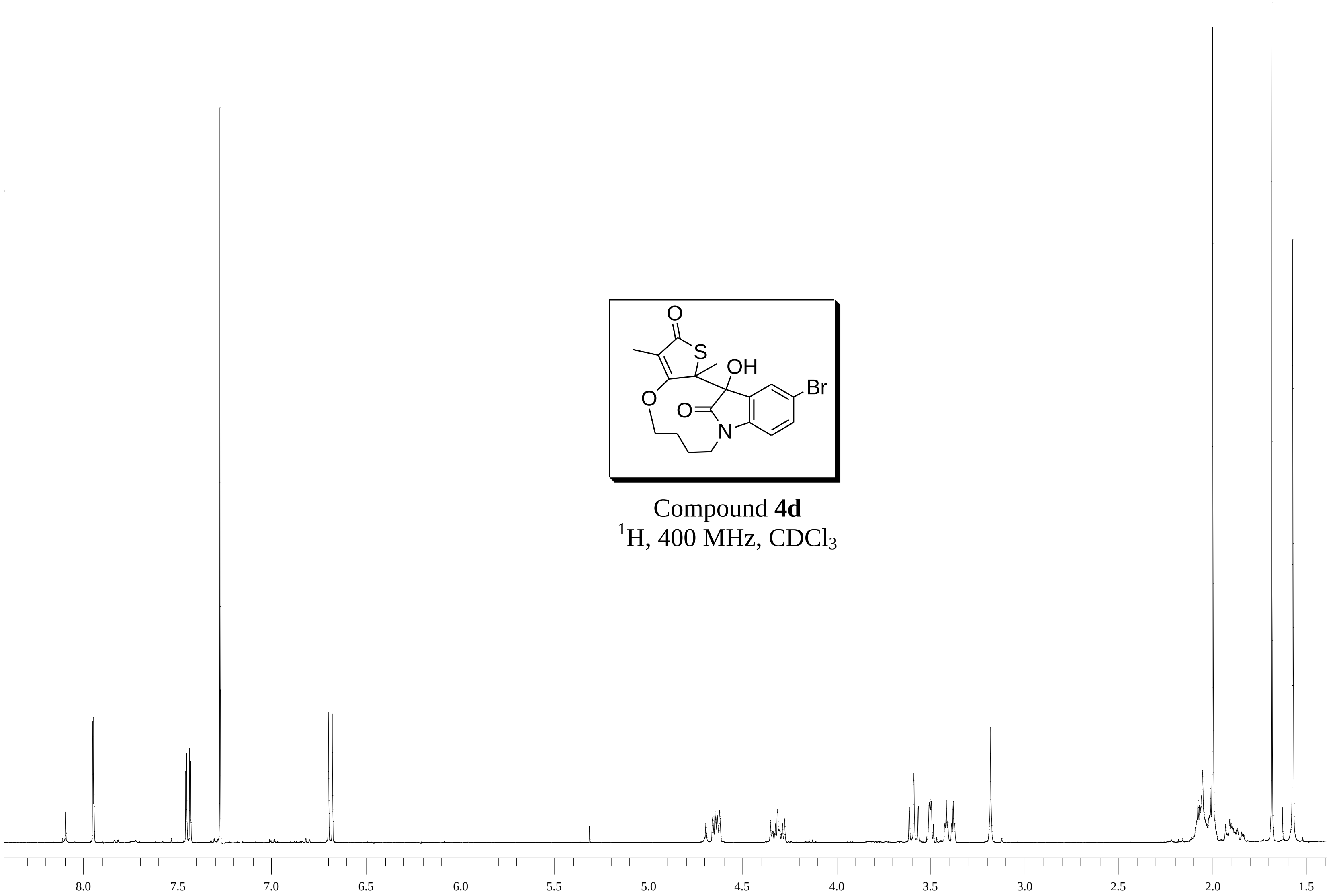

Compound **4d**
^{1}H, 400 MHz, $CDCl_3$

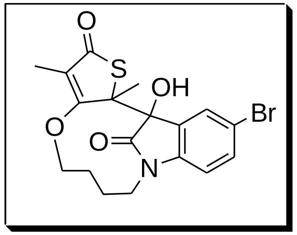

Compound **4d**
^{13}C, 75 MHz, $CDCl_3$

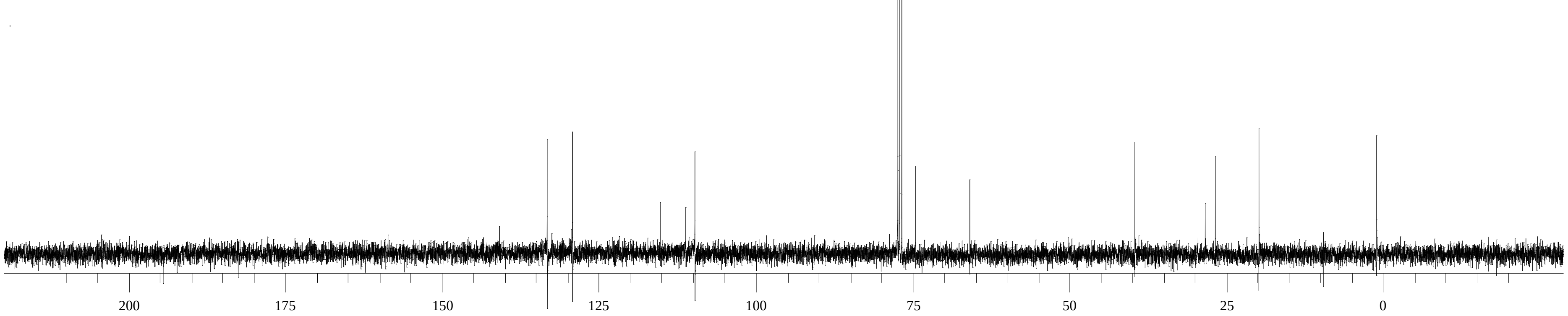

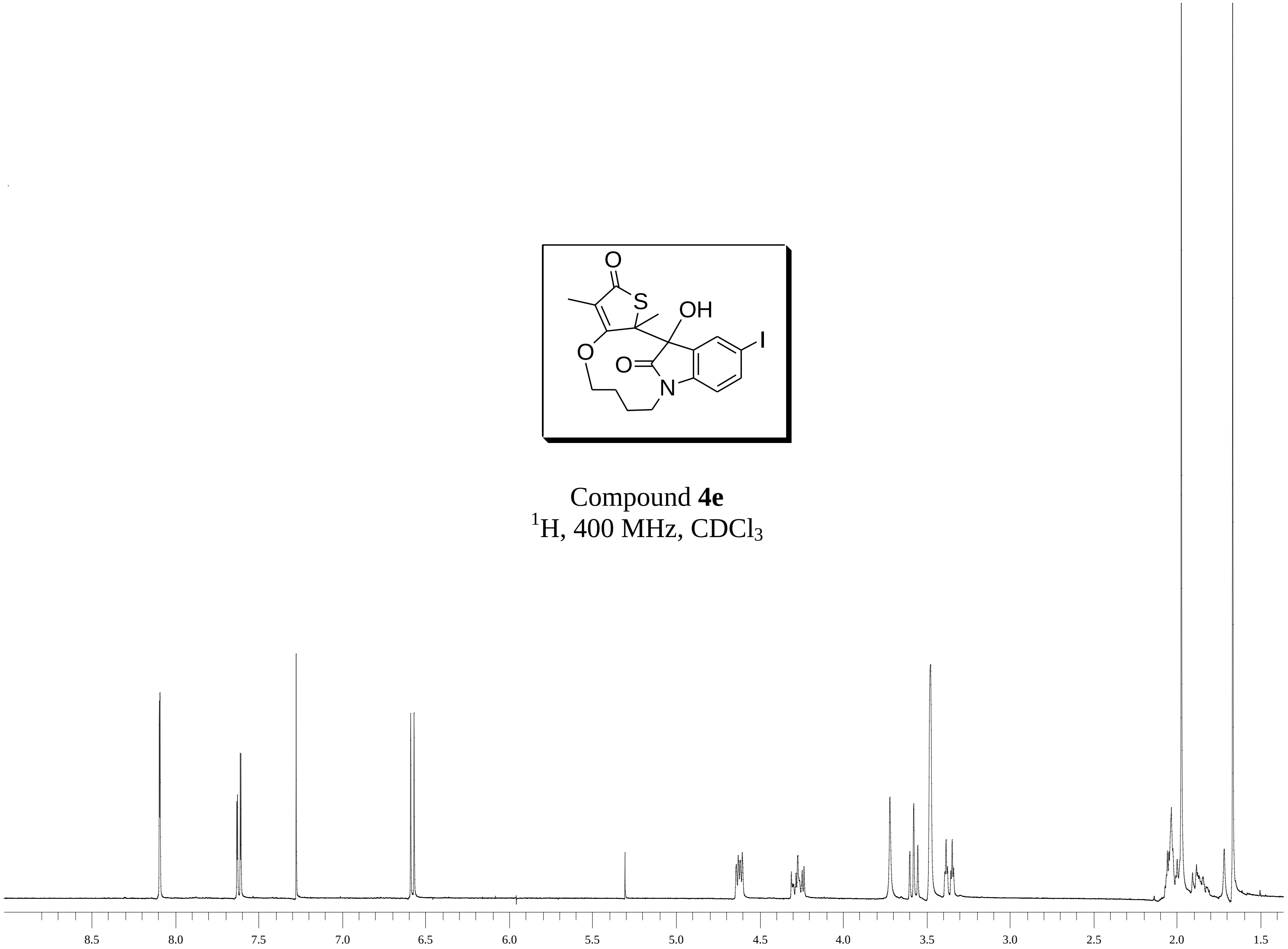

Compound **4e**
^{1}H, 400 MHz, $CDCl_3$

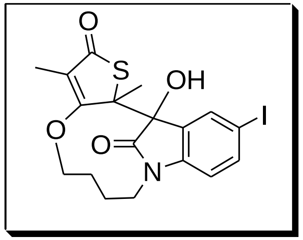

Compound **4e**
^{13}C, 100 MHz, $CDCl_3$

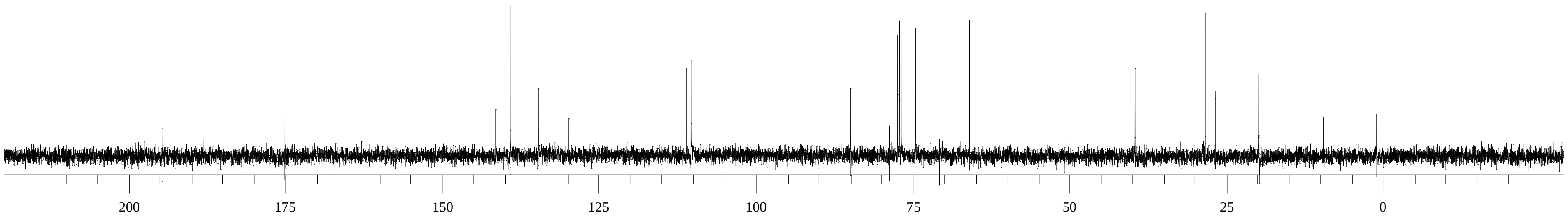

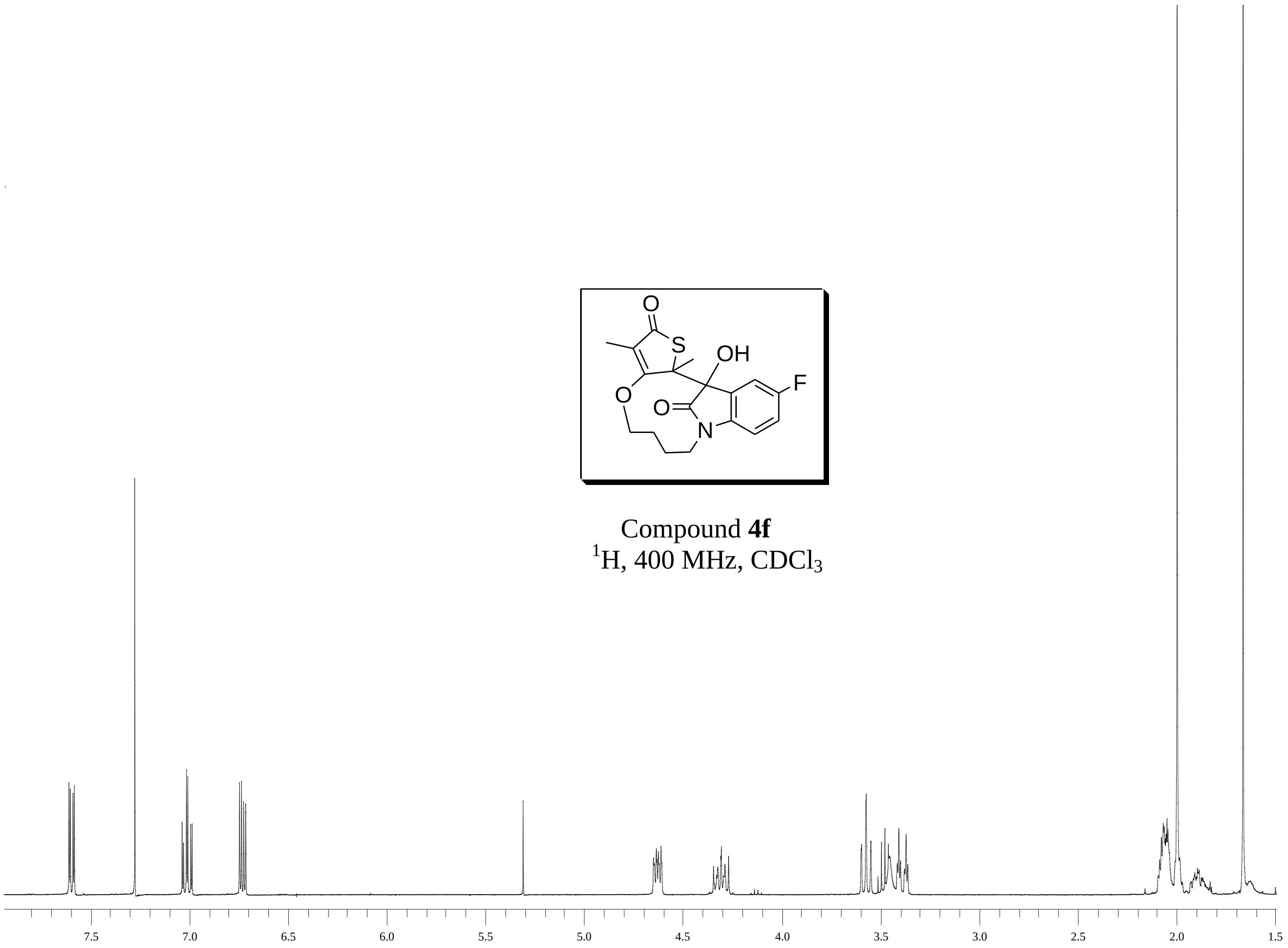

Compound **4f**
^{1}H, 400 MHz, $CDCl_3$

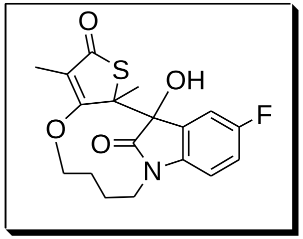

Compound **4f**

^{13}C, 100 MHz, $CDCl_3$

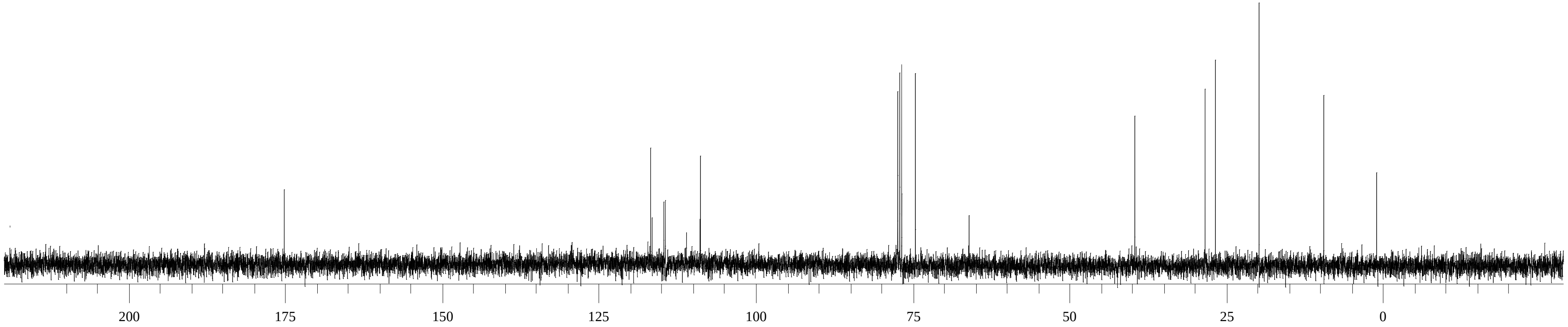

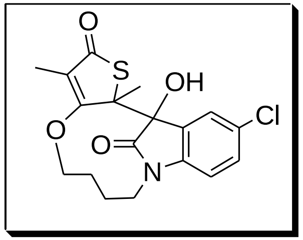

Compound **4g**

^{1}H, 400 MHz, DMSO-d_6

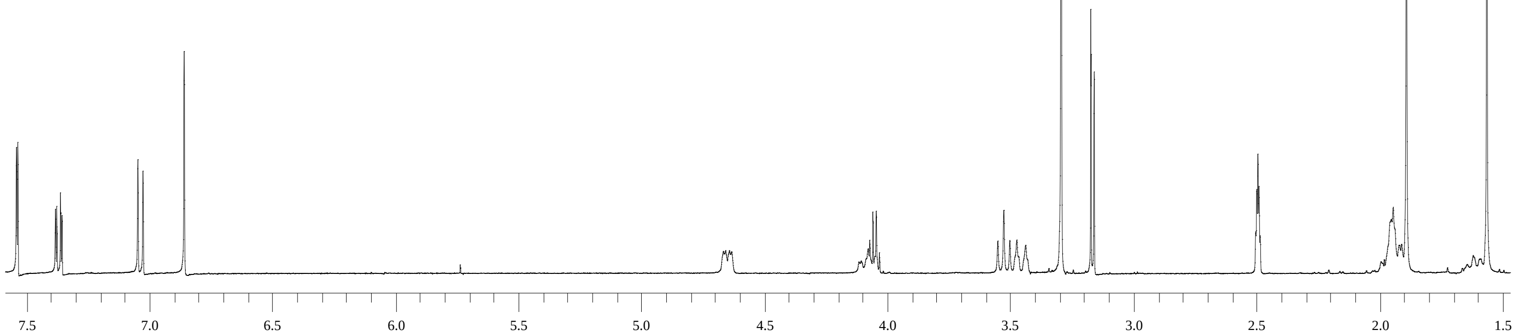

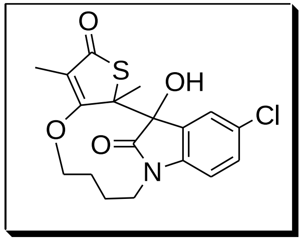

Compound **4g**

^{13}C, 100 MHz, DMSO-d_6

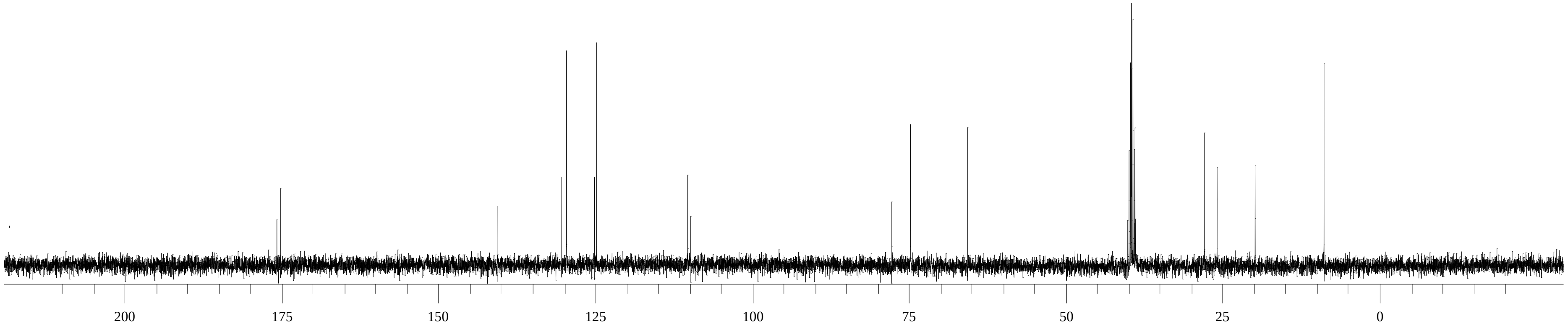

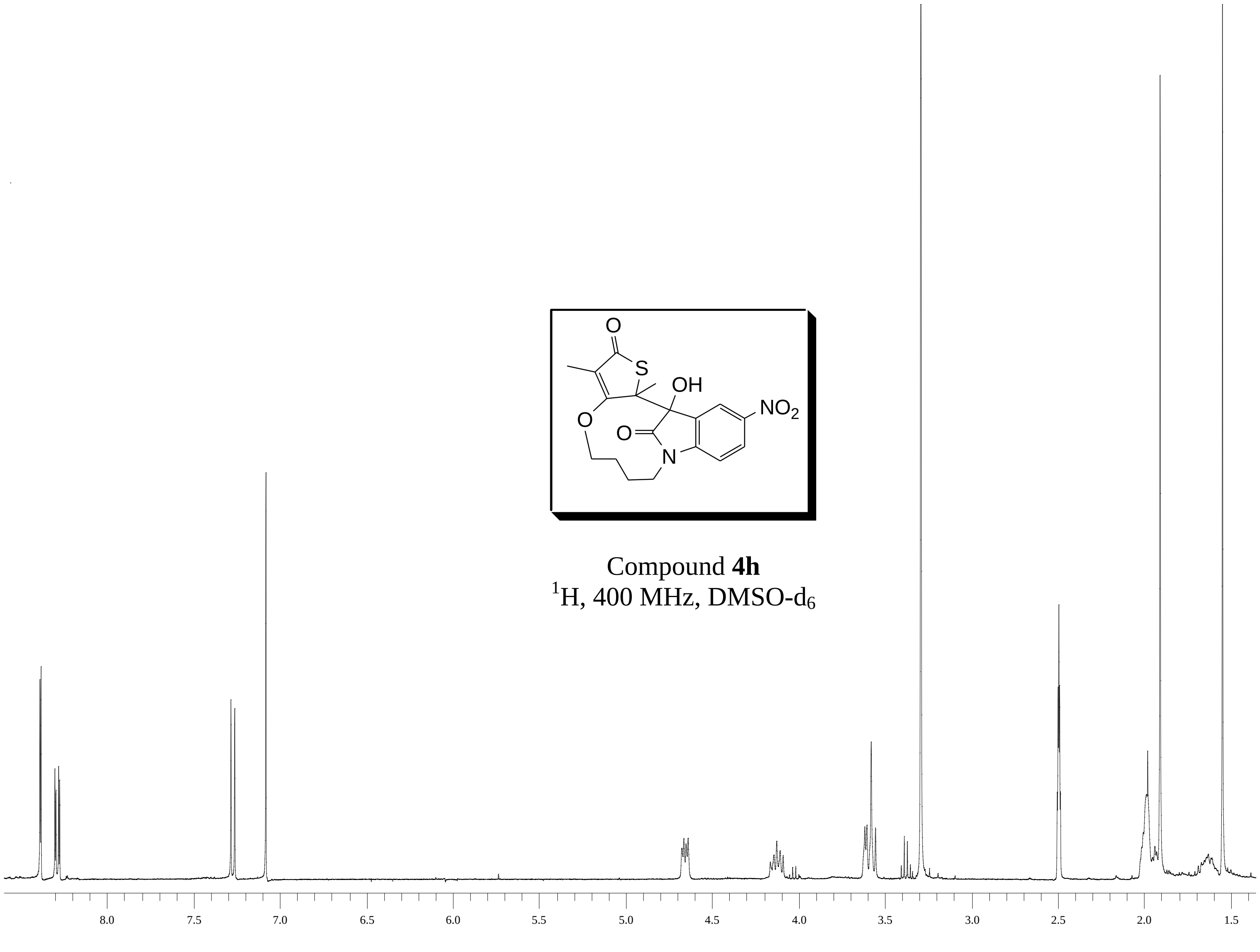

Compound **4h**
^{1}H, 400 MHz, DMSO-d_6

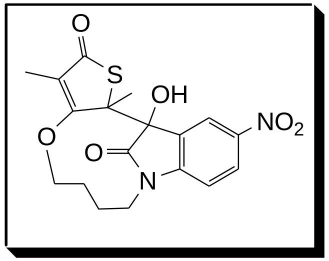

Compound **4h**
^{13}C, 100 MHz, DMSO-d$_6$

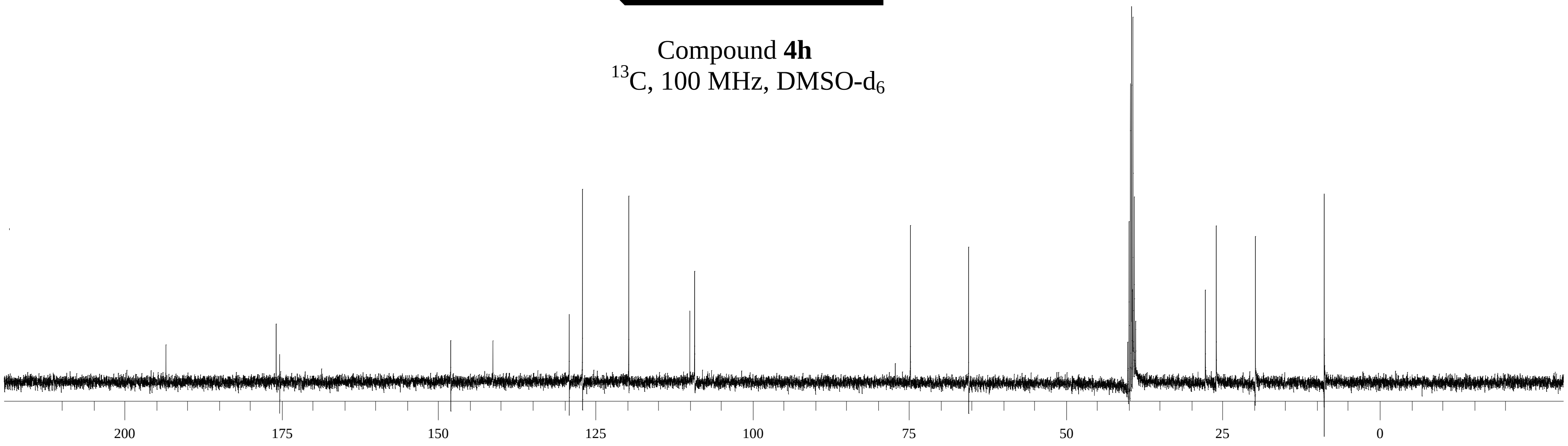

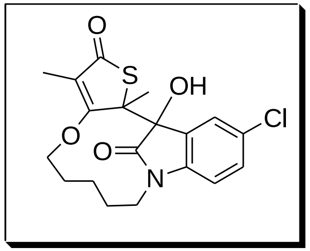

Compound **4i**
^{1}H, 300 MHz, $CDCl_3$

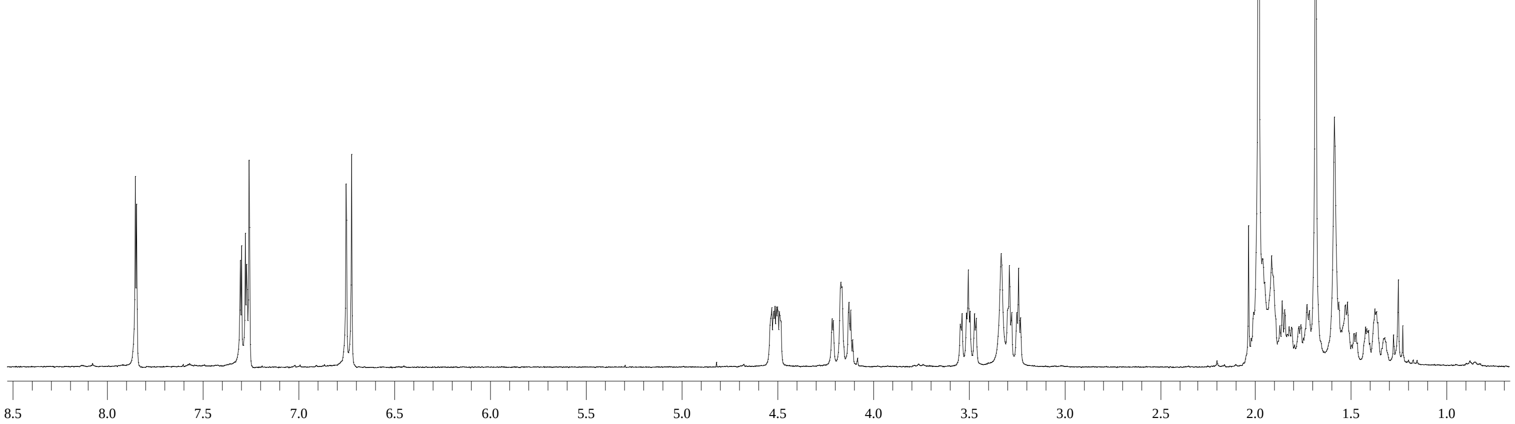

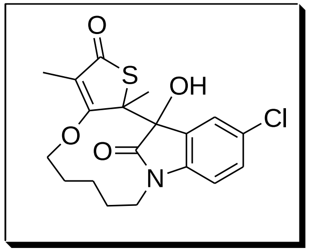

Compound **4i**
^{13}C, 75 MHz, $CDCl_3$

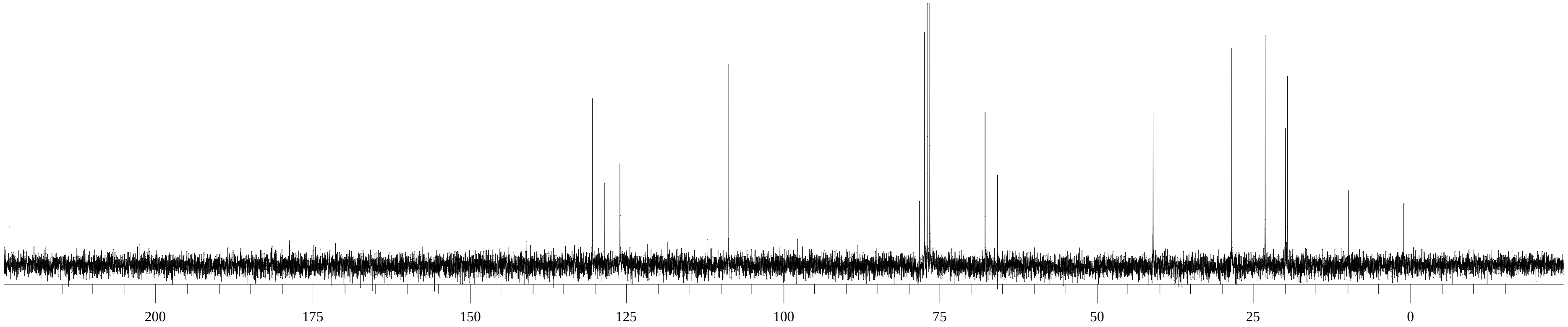

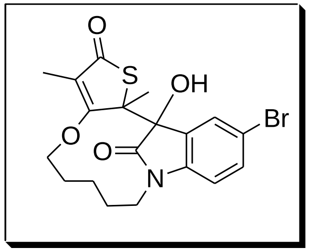

Compound **4j**
^{1}H, 300 MHz, $CDCl_3$

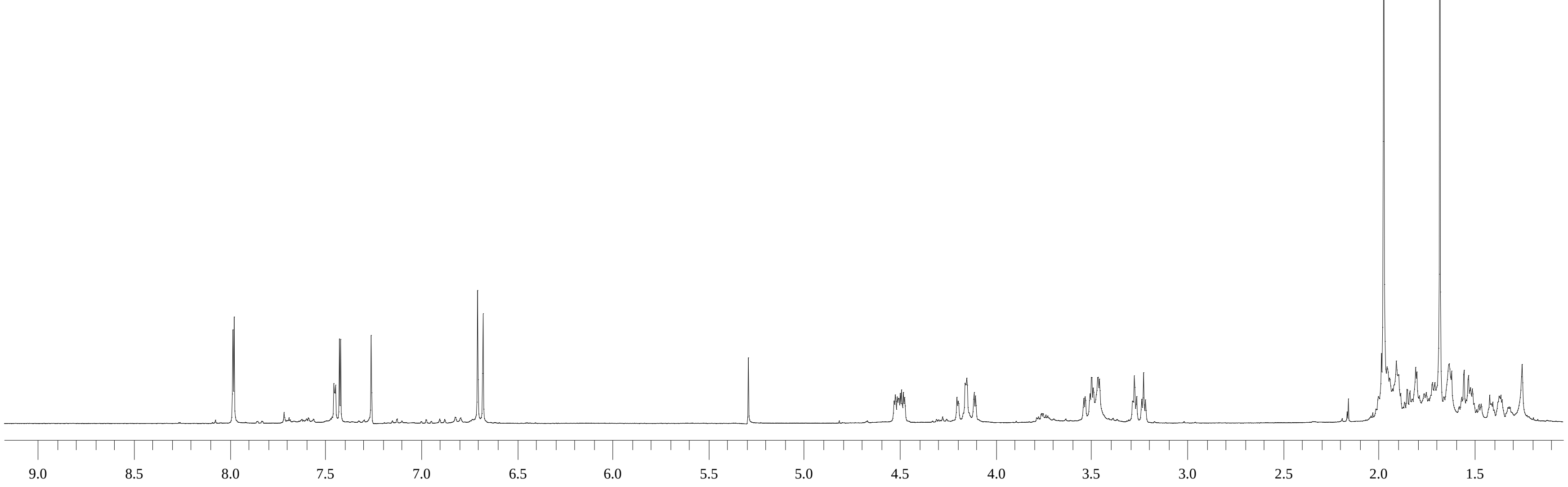

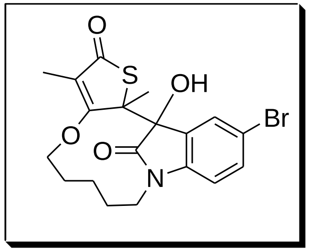

Compound **4j**
^{13}C, 75 MHz, $CDCl_3$

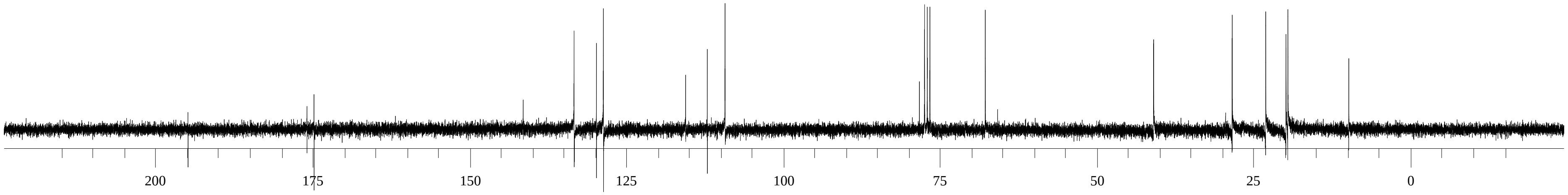

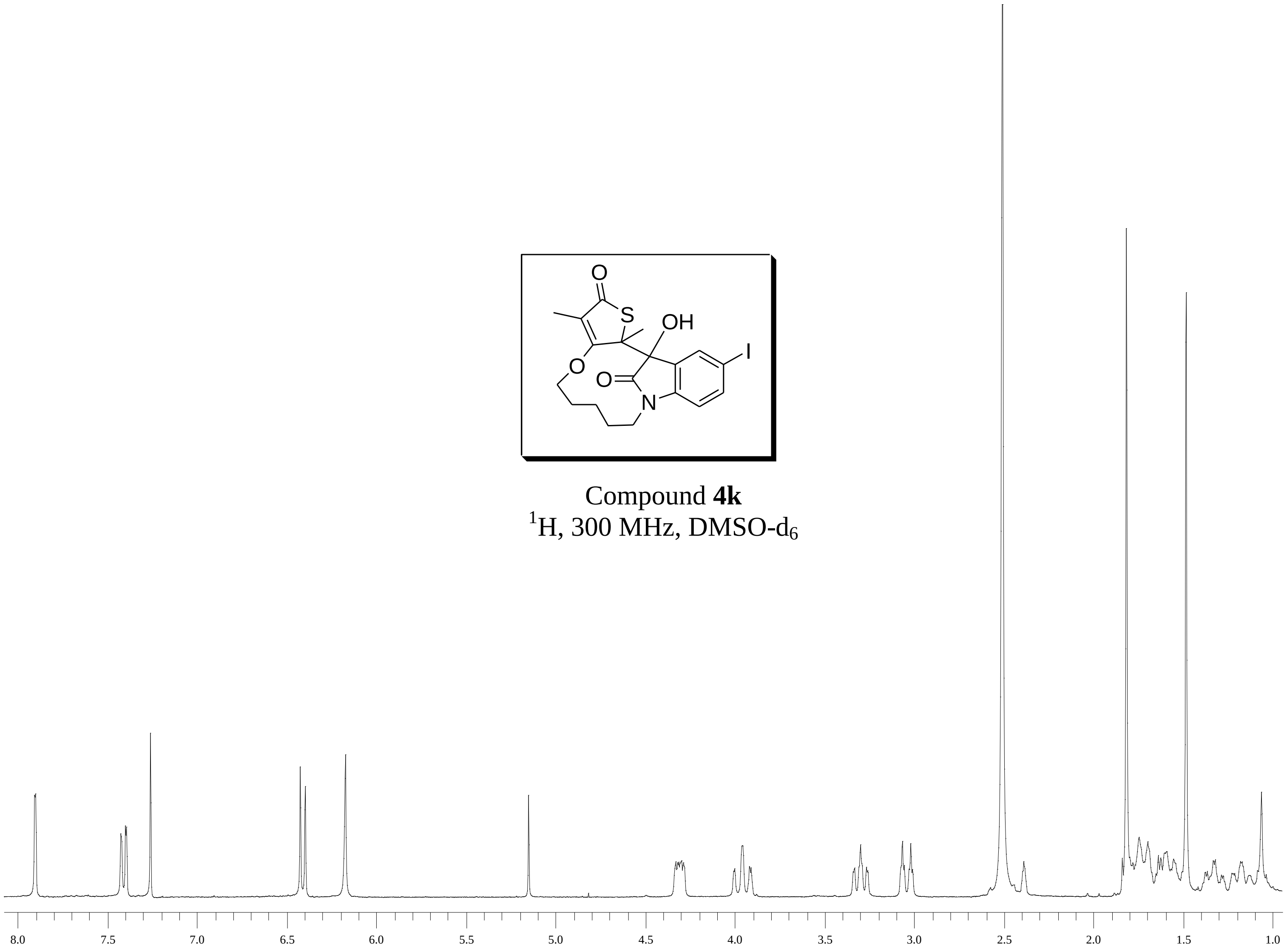

Compound **4k**
^{1}H, 300 MHz, DMSO-d_6

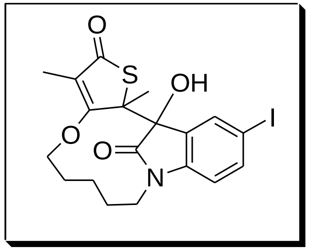

Compound **4k**
^{13}C, 75 MHz in DMSO-d_6

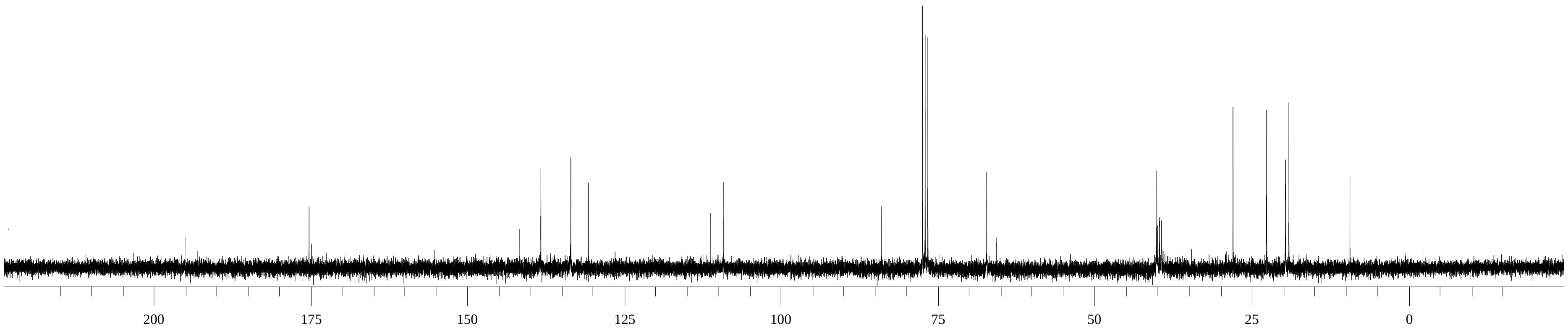

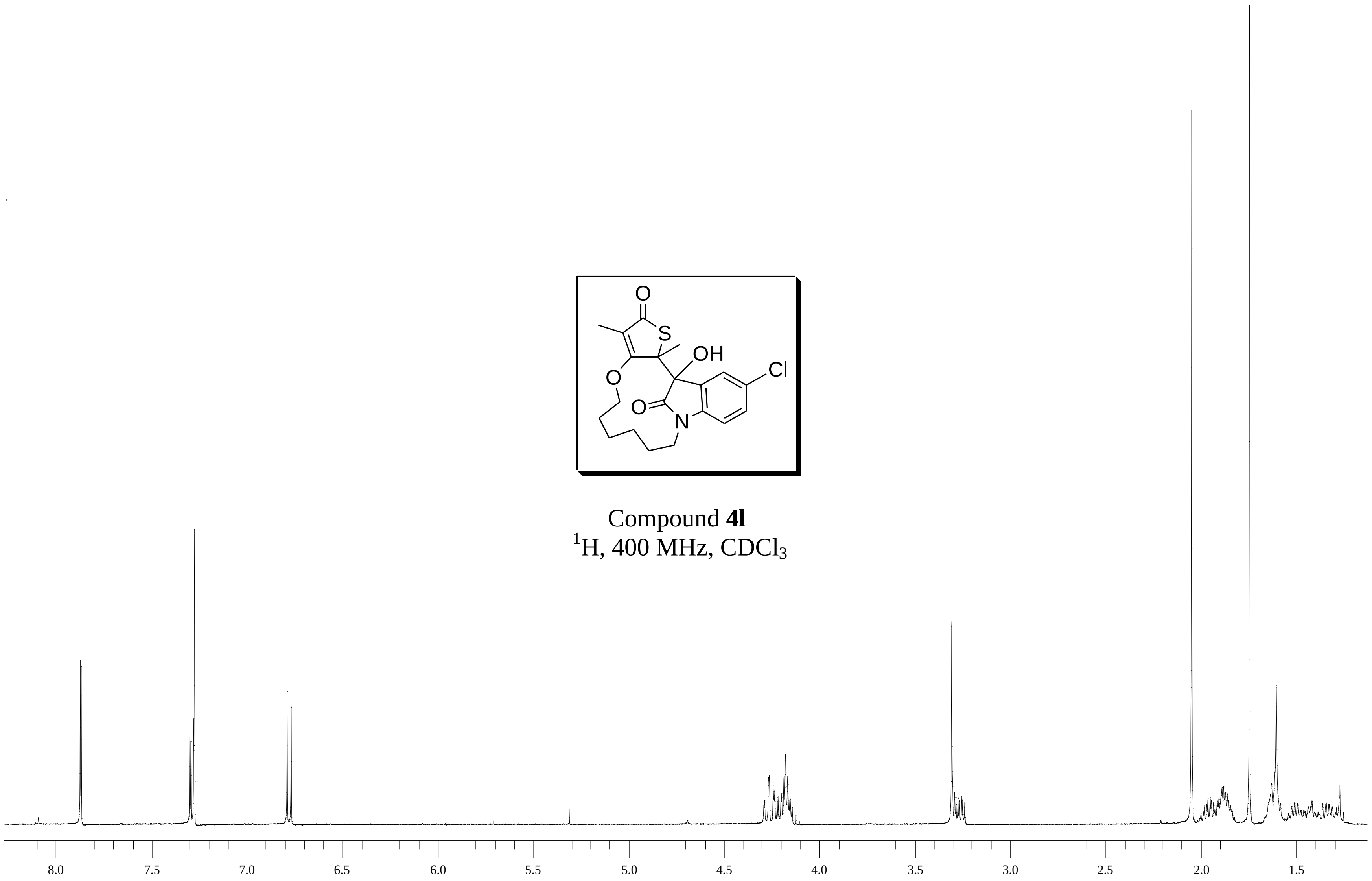
O
S
OH
Cl
O
O
N
Compound **4l**
^{1}H, 400 MHz, $CDCl_3$
8.0
7.5
7.0
6.5
6.0
5.5
5.0
4.5
4.0
3.5
3.0
2.5
2.0
1.5

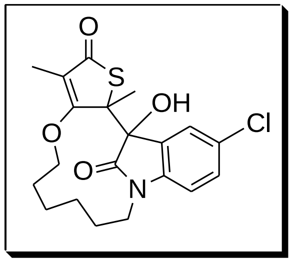

Compound **4l**

^{13}C, 100 MHz, $CDCl_3$

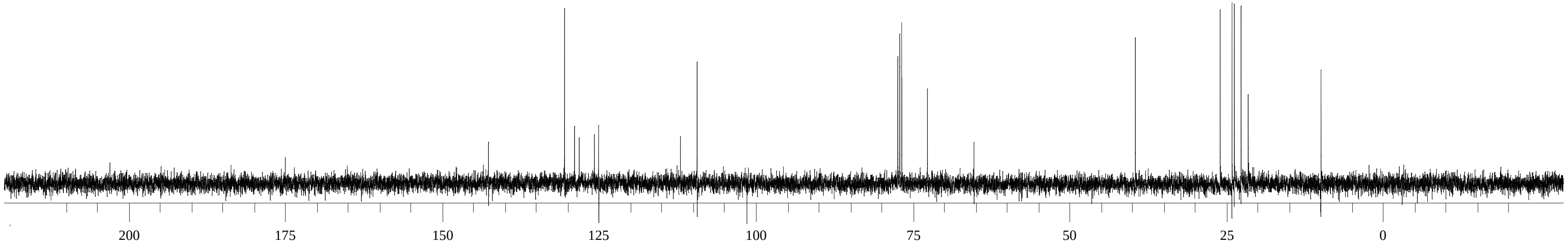

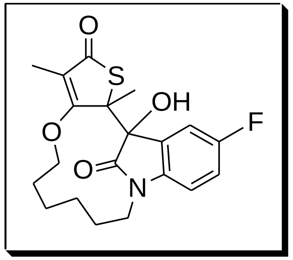

Compound **4m**

^{1}H, 400 MHz, $CDCl_3$

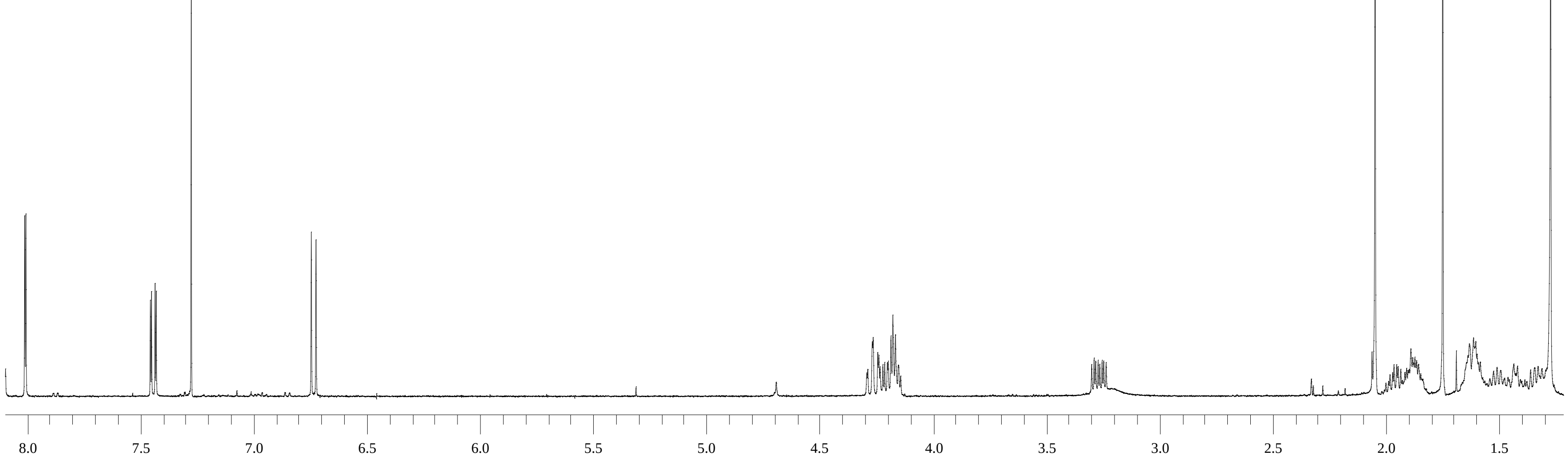

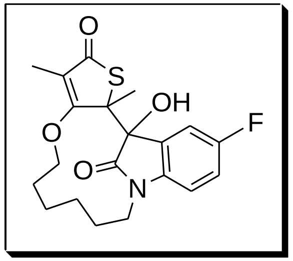

Compound **4m**
^{13}C, 100 MHz, $CDCl_3$

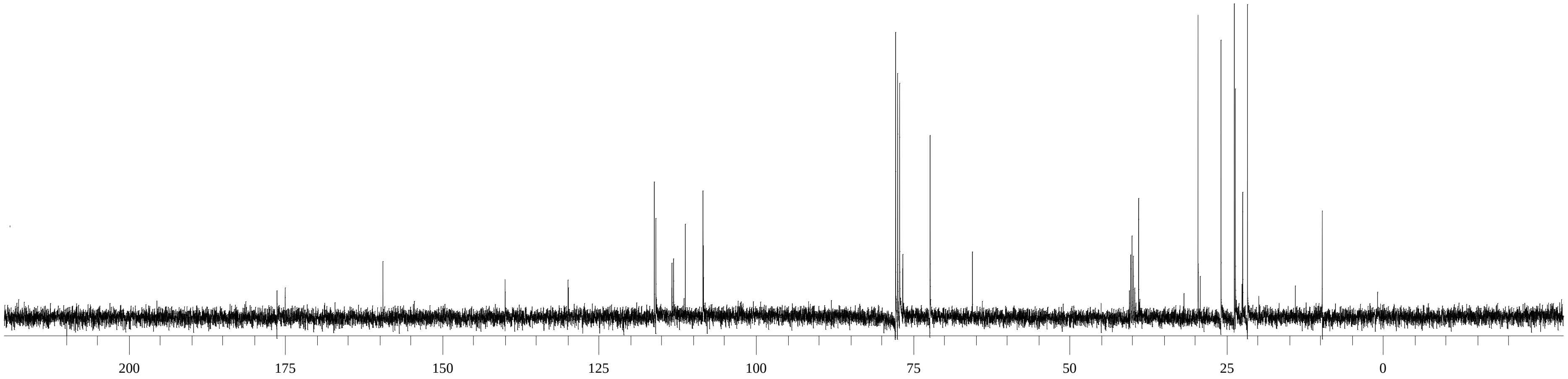

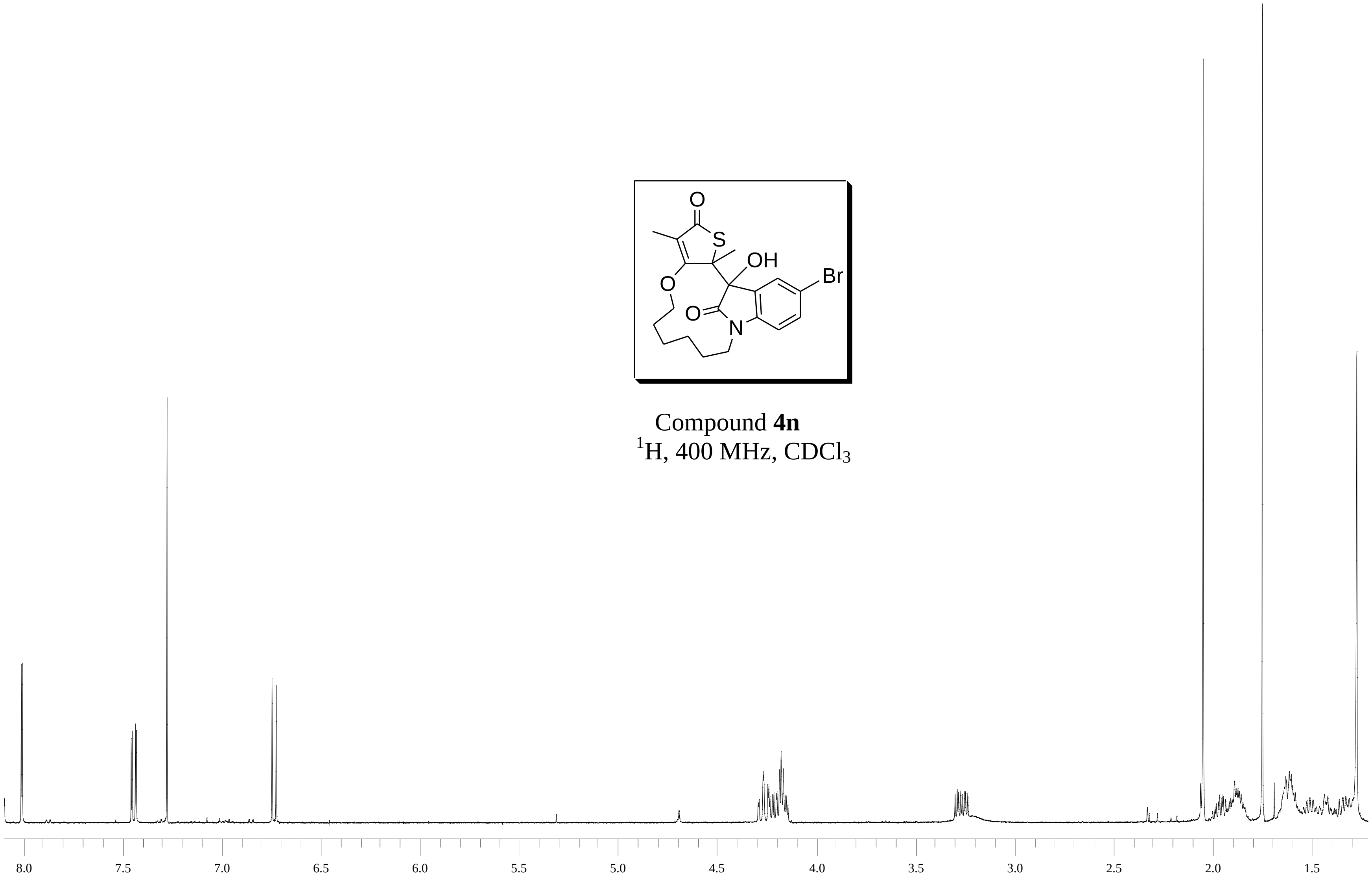

Compound **4n**
^{1}H, 400 MHz, $CDCl_3$

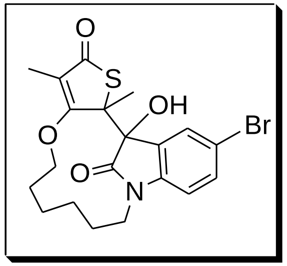

Compound **4n**
^{13}C, 100 MHz, $CDCl_3$

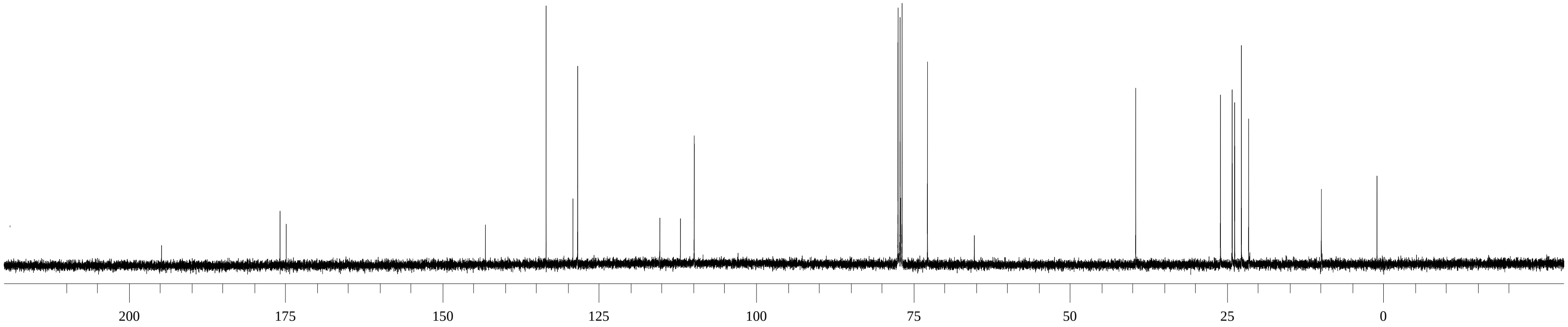

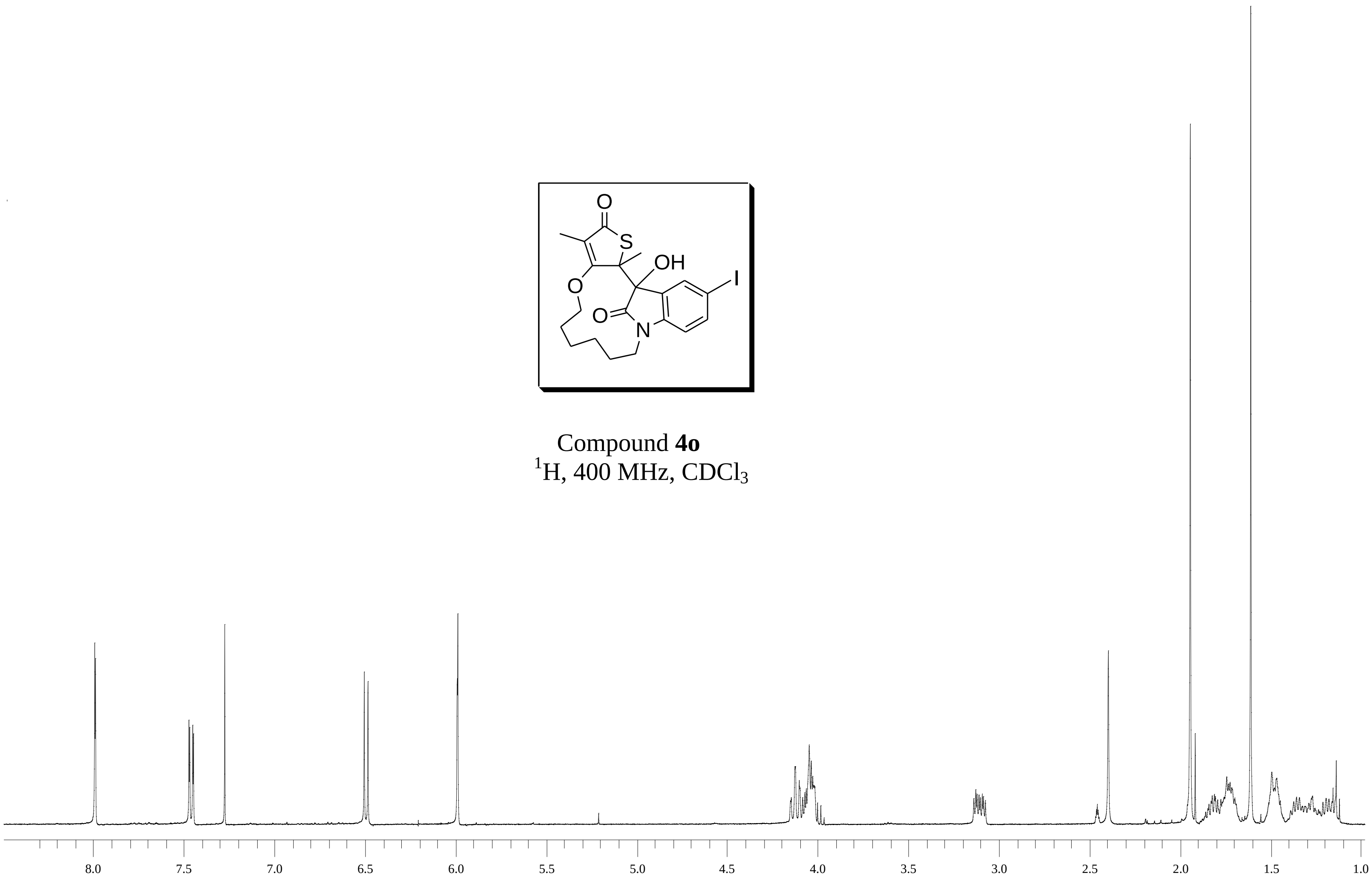

Compound **4o**
^{1}H, 400 MHz, $CDCl_3$

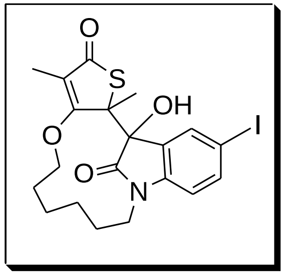

Compound **4o**
^{13}C, 100 MHz, $CDCl_3$

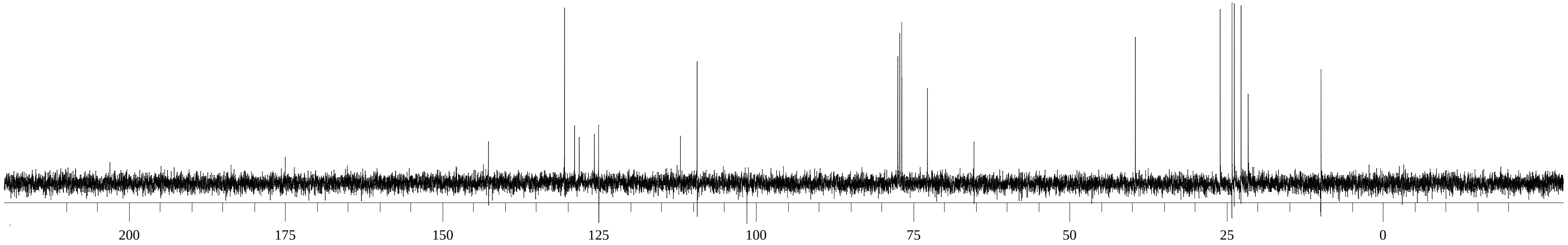

References

1. Perrin, D. D.; Armarego, W. L. F. *Purification of Laboratory Chemicals*: Pergamon Press, Oxford, 1988.
2. Otwinowski, Z.; Minor, W. In *Methods in Enzymology, Macromolecular Crystallography*; Carter Jr., C. W.; Sweet, R. M., Eds.; Academic Press, 1997; Vol. 276, pp 307–326.
3. Sheldrick, G. M. *SADABS*: University of Göttingen, Germany, 1996.
4. Sheldrick, G. M. *SHELXL-97 and SHELXS-97*: University of Göttingen, Germany, 1997.
5. Barbour, L. J. *J. Supramol. Chem.* **2001,** *1,* 189–191. doi:10.1016/S1472-7862(02)00030-8
6. Wang, C.-L. J.; Salvino, J. M. *Tetrahedron Lett.* **1984,** *25,* 5243–5246. doi:10.1016/S0040-4039(01)81574-4
